# Supplementary material for: The High-Affinity Interaction between ORC and DNA that Is Required for Replication Licensing Is Inhibited by 2-Arylquinolin-4-Amines
Source: Cell Chem Biol. 2017 Aug 17;24(8):981–992.e4. doi: 10.1016/j.chembiol.2017.06.019 (PMC5563080; doi:10.1016/j.chembiol.2017.06.019)
Supplement: Document S1. Figures S1–S6 [file mmc1.pdf]

Cell Chemical Biology, Volume *24*

Supplemental Information

The High-Affinity Interaction between ORC and DNA that Is Required for Replication Licensing Is Inhibited by 2-Arylquinolin-4-Amines

Nicola J. Gardner, Peter J. Gillespie, Jamie T. Carrington, Emma J. Shanks, Stuart P. McElroy, Emma J. Haagensen, Julie A. Frearson, Andrew Woodland, and J. Julian Blow

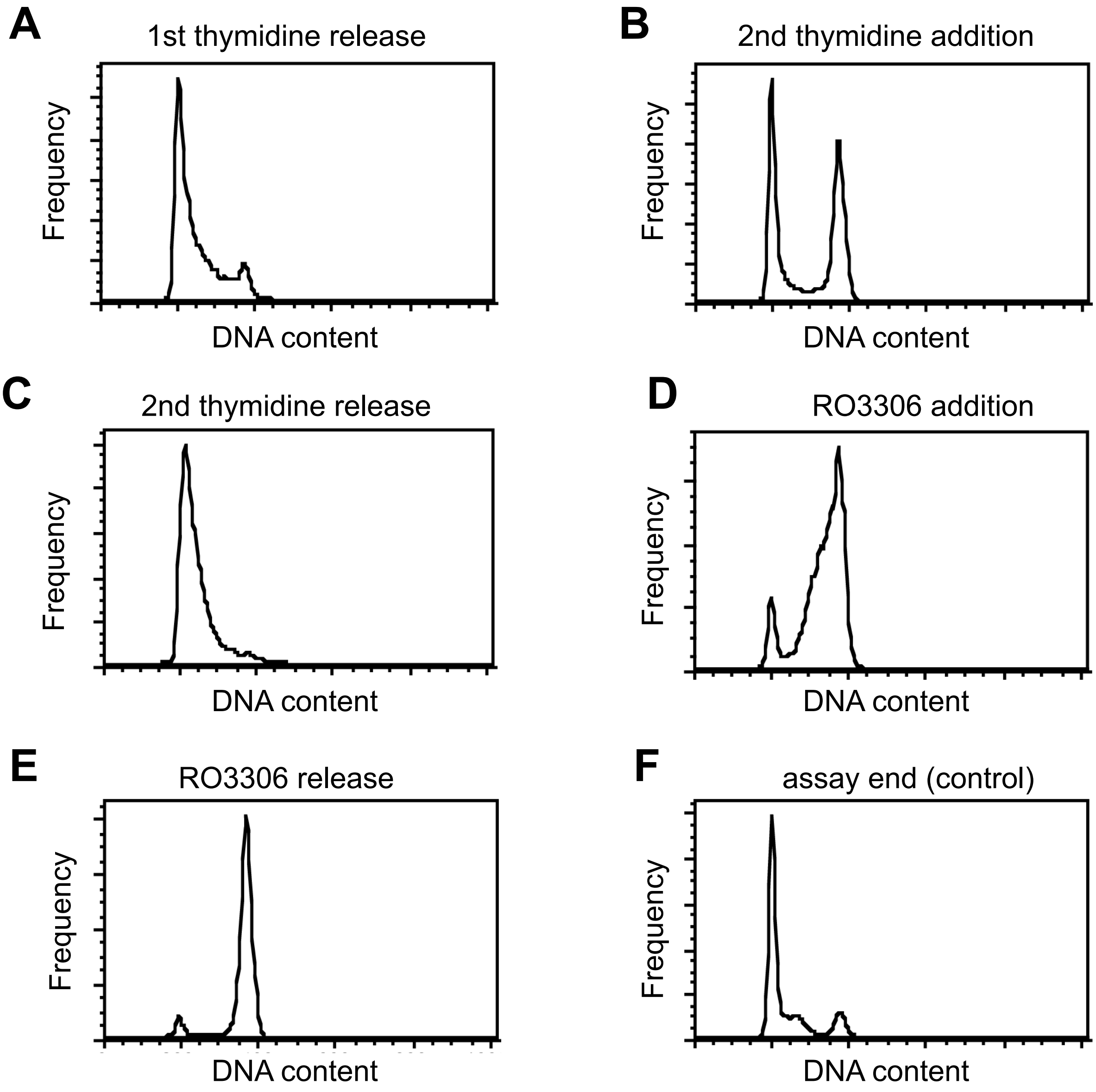

Supplementary Figure S1. Flow cytometry analysis of DNA content of cells at different stages in the screen (relates to Figure 1).

Cells were taken through the steps of the screen as shown in Figure 1A. At different steps, cells were isolated and stained with DAPI and their DNA content analysed by flow cytometry. **A.** Cells after the first 16 hr thymidine block. **B.** Cells 12 hr after release from the first thymidine block at the point when the 2nd thymidine block was applied. **C.** Cells at the end of the 2nd(12 hr) thymidine block. **D.** Cells 6 hr after release from the 2nd thymidine block, at the point when RO3306 was added. **E.** Cells after 10 hr RO3306 treatment, at the point of test compound addition. **F.** Cells 8 hr after release from the RO3306 block, at the point when the final Mcm4 assay was typically performed.

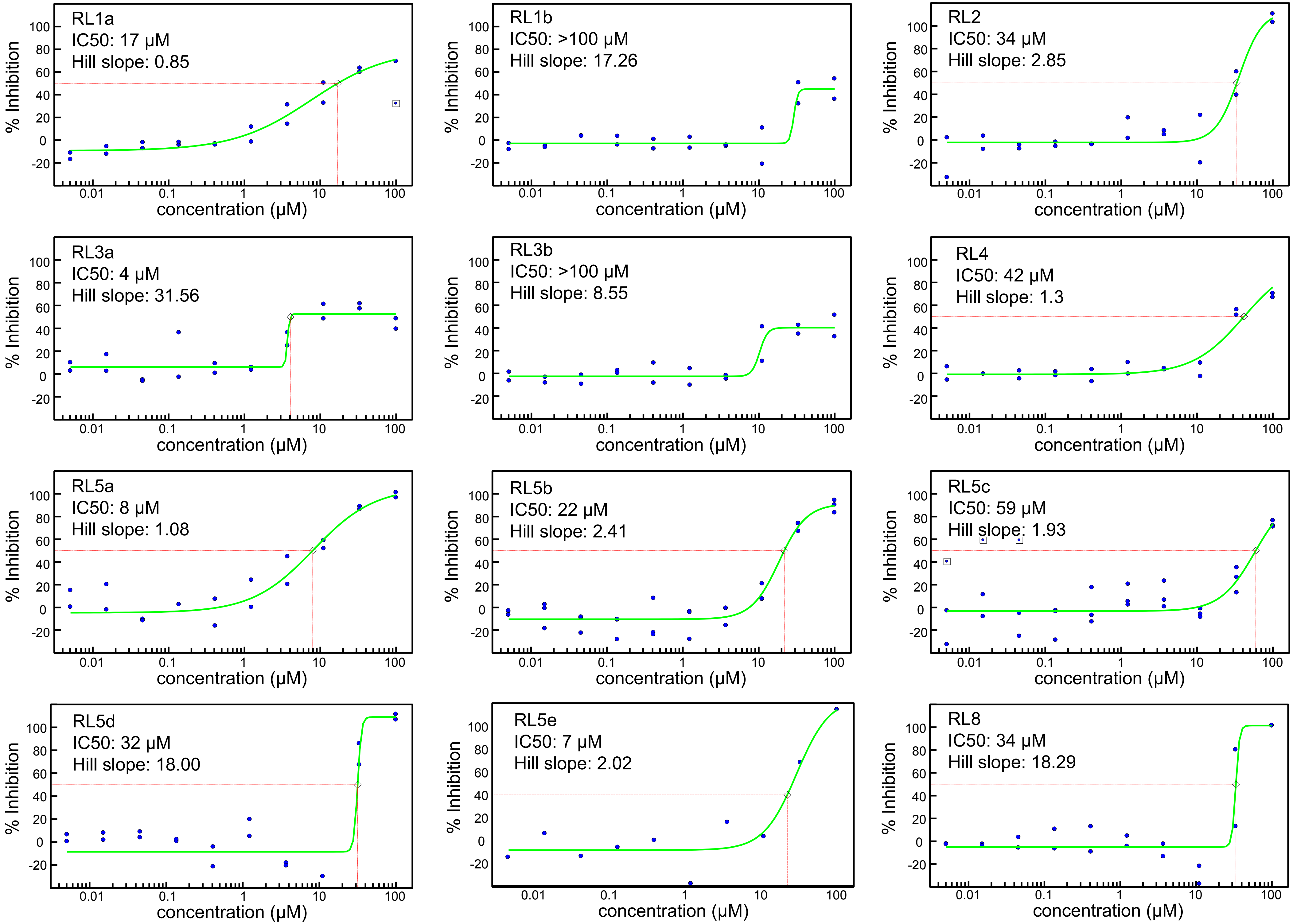

Supplementary Figure S2. Titration curves for selected compounds (relates to Figure 2).
12 compounds (RL1a-b, RL2, RL3a-b, RL4, RL5a-e and RL8) were selected after re-screening for a titration analysis. Assays for inhibition of Mcm4 DNA binding were performed in duplicate (apart from RL5e which was performed only once) and used to determine IC50 and Hill slope.

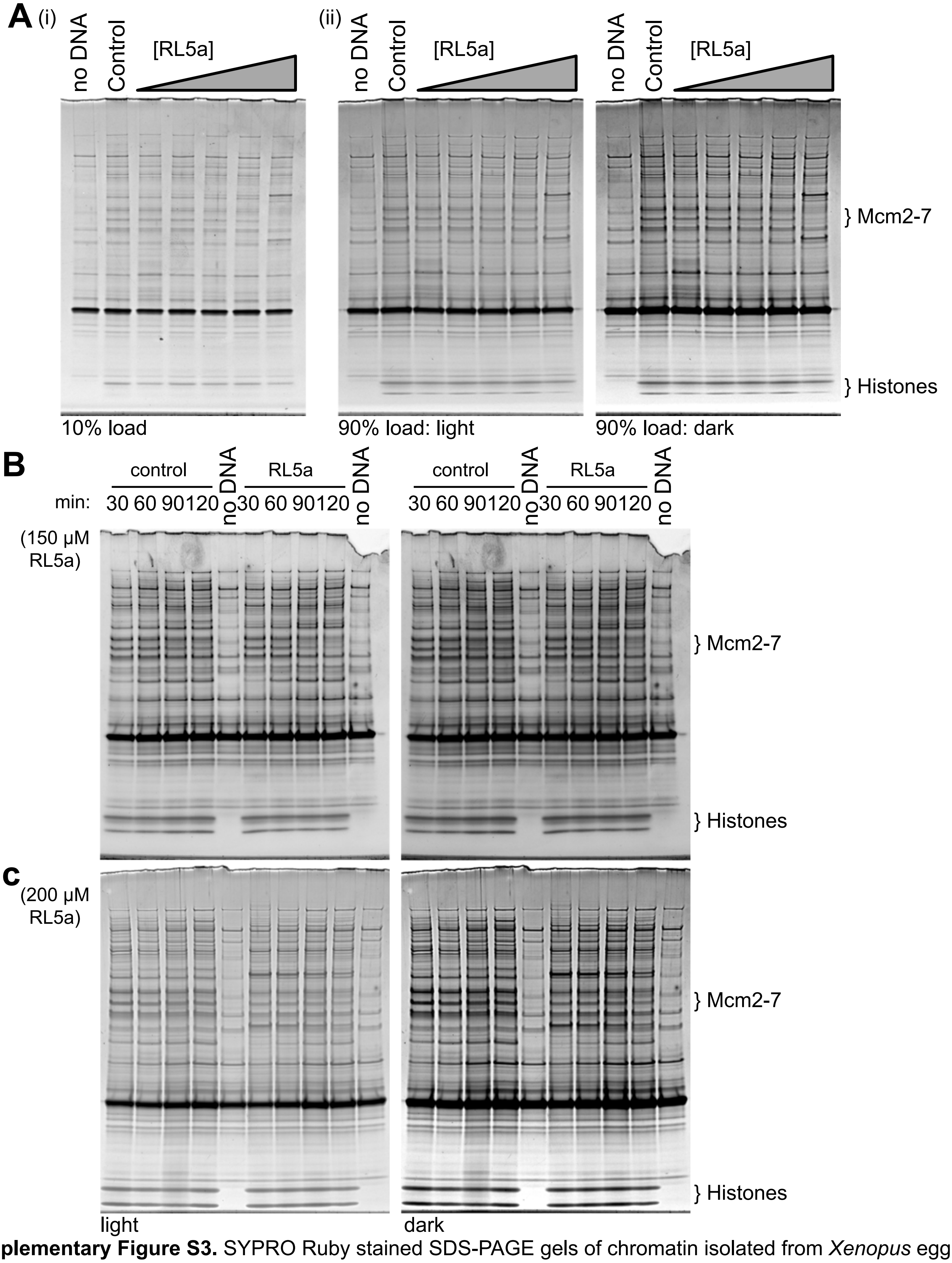

Supplementary Figure S3. SYPRO Ruby stained SDS-PAGE gels of chromatin isolated from *Xenopus* egg extract ± RL5a (relates to Figure 3).

Chromatin was isolated from *Xenopus* egg extract, supplemented or not with RL5a, at the indicated times, over a sucrose cushion. Isolated chromatin was subjected to SDS-PAGE and gels were stained with SYPRO Ruby to visualize recovered proteins. A 'no DNA' control was included to facilitate identification of chromatin associated proteins. **A.** Egg extract was supplemented with 25, 50, 100, 150 and 200 µM RL5a and chromatin was isolated at 30 min; (i) 10% load, (ii) 90% load, light and dark exposures. **B, C.** Egg extract was supplemented with RL5a at either (B) 150 µM or (C) 200 µM, and chromatin was isolated at the indicated times. Light and dark exposures of 90% load are shown.

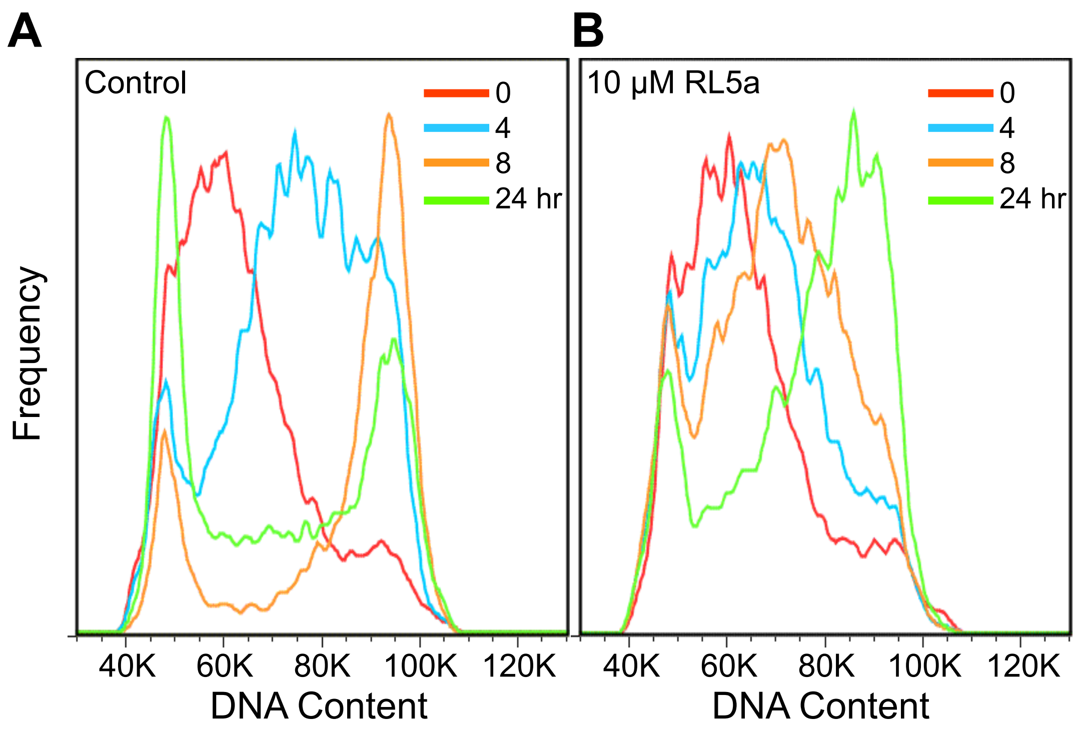

Supplementary Figure S4. Effect of RL5a after double-thymidine release (relates to Figure 3).
Following release from a double-thymidine synchronisation, in the absence (A) or presence (B) of 10 μM RL5a, DNA content (PI) of U2OS cells was measured by flow cytometry at the indicated times: 0 hr (red), 4 hr (blue), 8 hr (orange) and 24 hr (green).

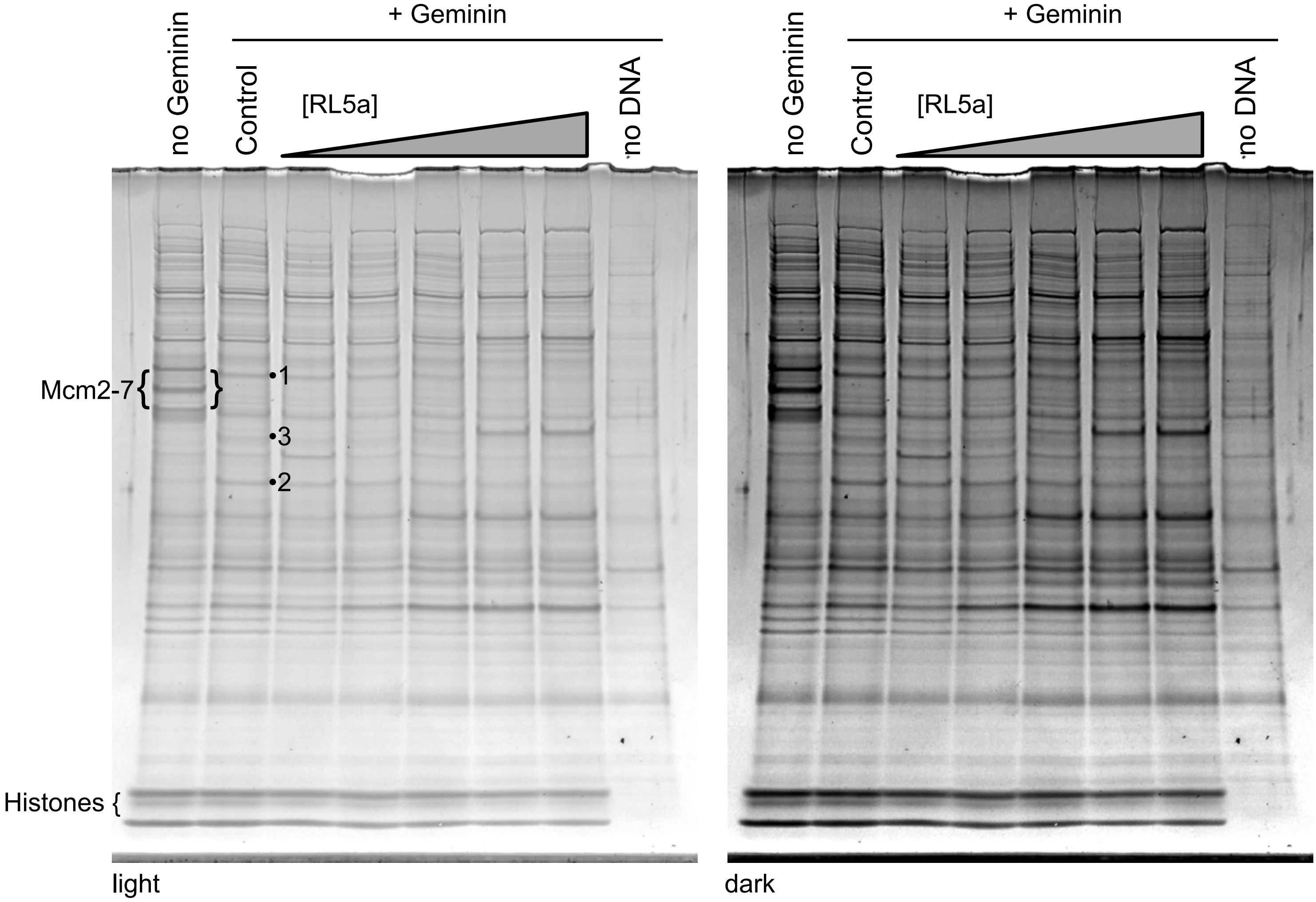

Supplementary Figure S5. SYPRO Ruby stained SDS-PAGE gel of chromatin isolated from geminin treated *Xenopus* egg extract ± RL5a (relates to Figure 5).
Chromatin was isolated from *Xenopus* egg extract, treated (or not) with geminin and supplemented (or not) with 25, 50, 100, 150 and 200 μM RL5a, over a sucrose cushion, at 20 min. Isolated chromatin was subjected to SDS-PAGE and the gel was stained with SYPRO Ruby to visualize recovered proteins. A 'no DNA' control was included to facilitate identification of chromatin associated proteins. Light and dark exposures are shown on the left and right respectively. The positions of Mcm2-7({}), Orc1 (•1), Orc2 (•2) and Orc3 (•3) are indicated.

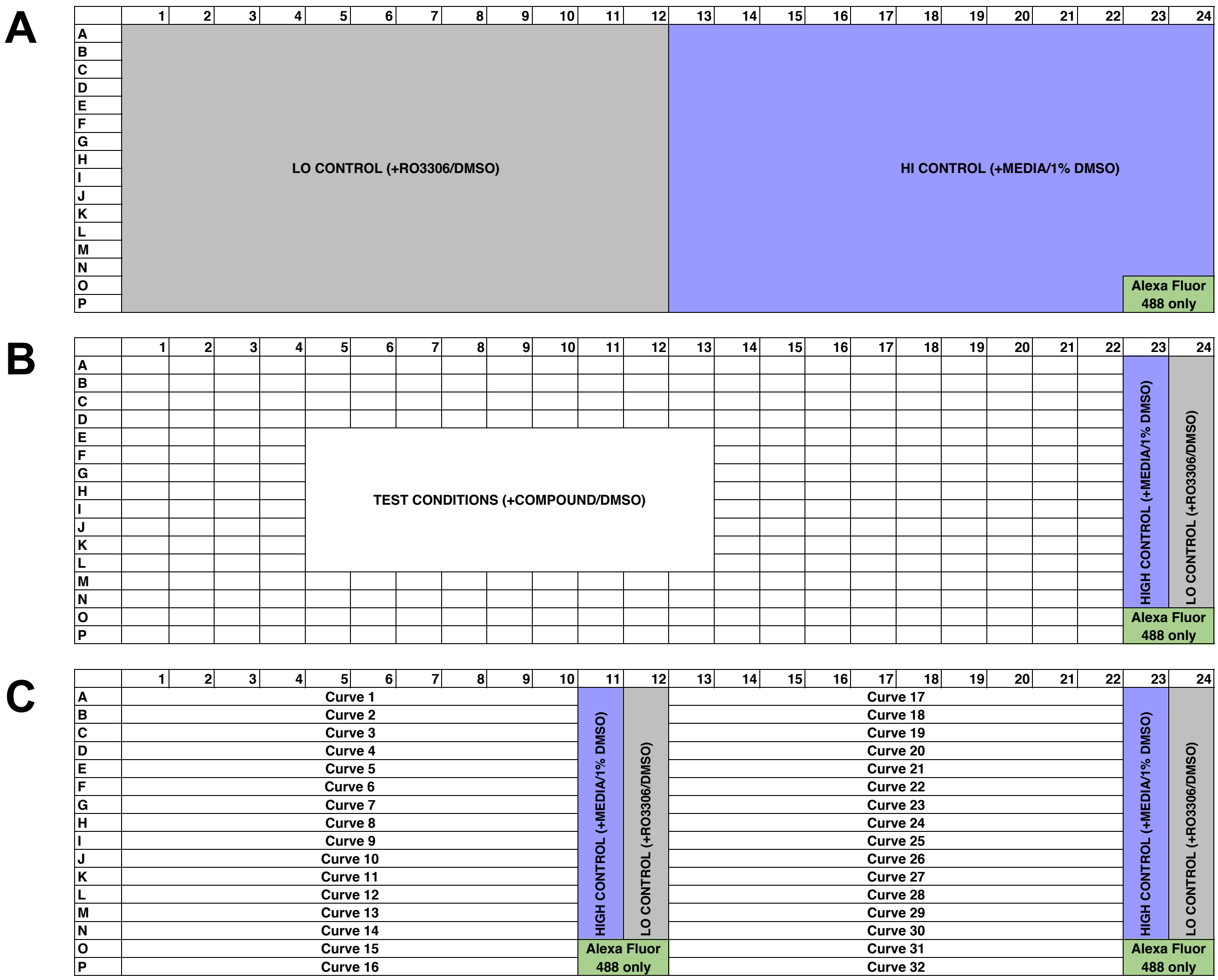

Supplementary Figure S6. Screening plate plans (relates to Figures 1 and 2). The plate plans for screening Quality Control plates (A), single point / duplicate point assay plates (B) and potency plates (C) are shown. Grey: Lo control (no release from RO-3306). Purple: Hi Control (release into G1 with DMSO alone). Green: Alexa Fluor 488 only (as Hi control, but no primary antibody). White: test conditions.
