# Supplementary material for: The High-Affinity Interaction between ORC and DNA that Is Required for Replication Licensing Is Inhibited by 2-Arylquinolin-4-Amines
Source: Cell Chem Biol. 2017 Aug 17;24(8):981–992.e4. doi: 10.1016/j.chembiol.2017.06.019 (PMC5563080; doi:10.1016/j.chembiol.2017.06.019)
Supplement: Document S2. Article plus Supplemental Information [file mmc2.pdf]

Article

Cell Chemical Biology

# The High-Affinity Interaction between ORC and DNA that Is Required for Replication Licensing Is Inhibited by 2-Arylquinolin-4-Amines

## Graphical Abstract

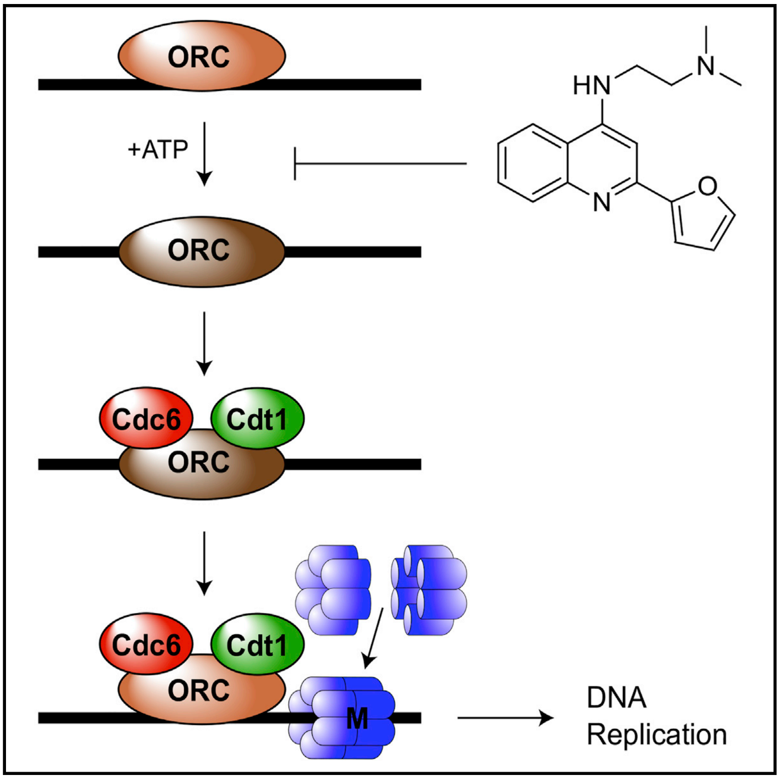


## Highlights

- Arylquinolin-amines identified as small-molecule inhibitors of replication licensing
- Inhibitor prevents tight ORC-DNA interaction required for MCM2–7 loading
- ORC-DNA binding inhibited allosterically to mimic a lack of ATP

## Authors

Nicola J. Gardner, Peter J. Gillespie, Jamie T. Carrington, ..., Julie A. Frearson, Andrew Woodland, J. Julian Blow

## Correspondence

a.woodland@dundee.ac.uk (A.W.), j.j.blow@dundee.ac.uk (J.J.B.)

## In Brief

Gardner et al. describe the identification of a family of arylquinolin-amines as inhibitors of "replication licensing", the loading of replication origins with double hexamers of MCM2–7. Arylquinolin-amines prevent the tight ORC-DNA interaction required for licensing, mimicking a lack of ATP.

Gardner et al., 2017, Cell Chemical Biology *24*, 981–992
August 17, 2017 © 2017 The Authors. Published by Elsevier Ltd.
http://dx.doi.org/10.1016/j.chembiol.2017.06.019

CellPress

# The High-Affinity Interaction between ORC and DNA that Is Required for Replication Licensing Is Inhibited by 2-Arylquinolin-4-Amines

Nicola J. Gardner,[1,3] Peter J. Gillespie,[1,3] Jamie T. Carrington,[1] Emma J. Shanks,[2,4] Stuart P. McElroy,[2] Emma J. Haagensen,[1,5] Julie A. Frearson,[2,6] Andrew Woodland,[2,*] and J. Julian Blow[1,7,*]
[1]Centre for Gene Regulation & Expression, School of Life Sciences, University of Dundee, Dundee DD1 5EH, UK
[2]Drug Discovery Unit, School of Life Sciences, University of Dundee, Dundee DD1 5EH, UK
[3]These authors contributed equally
[4]Present address: Cancer Research UK Beatson Institute, Glasgow G61 1BD, UK
[5]Present address: Northern Institute for Cancer Research, Medical School, Newcastle University, Newcastle upon Tyne NE2 4HH, UK
[6]Present address: Charles River Laboratories International Inc, Wilmington, MA 01887, USA
[7]Lead Contact
*Correspondence: a.woodland@dundee.ac.uk (A.W.), j.j.blow@dundee.ac.uk (J.J.B.)
http://dx.doi.org/10.1016/j.chembiol.2017.06.019

## SUMMARY

**In late mitosis and $G_1$, origins of DNA replication must be "licensed" for use in the upcoming S phase by being encircled by double hexamers of the minichromosome maintenance proteins MCM2–7. A "licensing checkpoint" delays cells in $G_1$ until sufficient origins have been licensed, but this checkpoint is lost in cancer cells. Inhibition of licensing can therefore kill cancer cells while only delaying normal cells in $G_1$. In a high-throughput cell-based screen for licensing inhibitors we identified a family of 2-arylquinolin-4-amines, the most potent of which we call RL5a. The binding of the origin recognition complex (ORC) to origin DNA is the first step of the licensing reaction. We show that RL5a prevents ORC forming a tight complex with DNA that is required for MCM2–7 loading. Formation of this ORC-DNA complex requires ATP, and we show that RL5a inhibits ORC allosterically to mimic a lack of ATP.**

## INTRODUCTION

During S phase of the eukaryotic cell division cycle, pairs of replication forks are initiated at replication origins distributed throughout the genome. These replication origins must be regulated so that during each cell cycle no sections of DNA are left unreplicated and no sections of DNA are replicated more than once. Eukaryotes achieve this by dividing the replication process into two non-overlapping phases. During late mitosis and early $G_1$, replication origins are "licensed" for future use by being loaded with double hexamers of the MCM2–7 proteins (Blow and Dutta, 2005; Arias and Walter, 2007). Each MCM2–7 hexamer forms a ring with a positively charged central channel. In the licensing reaction, the MCM2–7 hexamers are clamped around double-stranded DNA (Evrin et al., 2009; Remus et al., 2009; Gambus et al., 2011). During S phase, the Cdc45 and GINS proteins associate with the MCM2–7 double hexamers to form the replicative CMG (Cdc45-MCM-GINS) helicase (Moyer et al., 2006; Ilves et al., 2010). Since the ability to license replication origins ceases before cells enter S phase, MCM2–7 hexamers are exclusively associated with unreplicated DNA, thereby preventing re-replication of DNA (Blow and Dutta, 2005; Arias and Walter, 2007).

However, individual forks can irreversibly stall before termination. This can be caused by forks encountering DNA damage or tightly associated DNA-protein complexes, or by fork movement being slowed by replication inhibitors (Lambert and Carr, 2005). If two converging replication forks irreversibly stall, there is no simple way for the cell to replicate the intervening DNA. Cells cannot load new MCM2–7 hexamers onto the DNA between the two stalled forks, as this would also allow the reloading of MCM2–7 onto replicated DNA, leading to re-replication. Cells protect themselves from the consequences of irreversible fork stalling by licensing an oversufficiency of replication origins, not all of which are used in any given S phase, with the majority instead remaining dormant (Woodward et al., 2006; Ge et al., 2007; Ibarra et al., 2008; Blow and Ge, 2009; Ge and Blow, 2010; Blow et al., 2011).

Because there is no opportunity to license new origins once cells have entered S phase, it is critical that cells exit $G_1$ only when they have licensed a sufficient number of origins. To enforce this many cells possess a "licensing checkpoint" that delays progression out of $G_1$ until enough origins have been licensed (Shreeram et al., 2002; Feng et al., 2003; Montagnoli et al., 2004; Machida et al., 2005; Teer et al., 2006; Liu et al., 2009; Blow and Gillespie, 2008; Nevis et al., 2009). It is currently unclear precisely how the licensing checkpoint assesses the number of licensed origins. When the checkpoint is engaged it suppresses CDK activity that is required for cells to progress out of $G_1$ into S phase (Shreeram et al., 2002; Machida et al., 2005; Teer et al., 2006; Liu et al., 2009; Nevis et al., 2009). This downregulation of CDK activity likely occurs by several mechanisms, including suppression of cyclin D1 transcription (Liu et al., 2009), decreased levels of cyclin E (Shreeram et al., 2002; Teer et al., 2006), inhibition of essential CDK2 phosphorylation on threonine 160 (Nevis et al., 2009), and the activation of


© 2017 The Authors. Published by Elsevier Ltd.
This is an open access article under the CC BY license (http://creativecommons.org/licenses/by/4.0/).

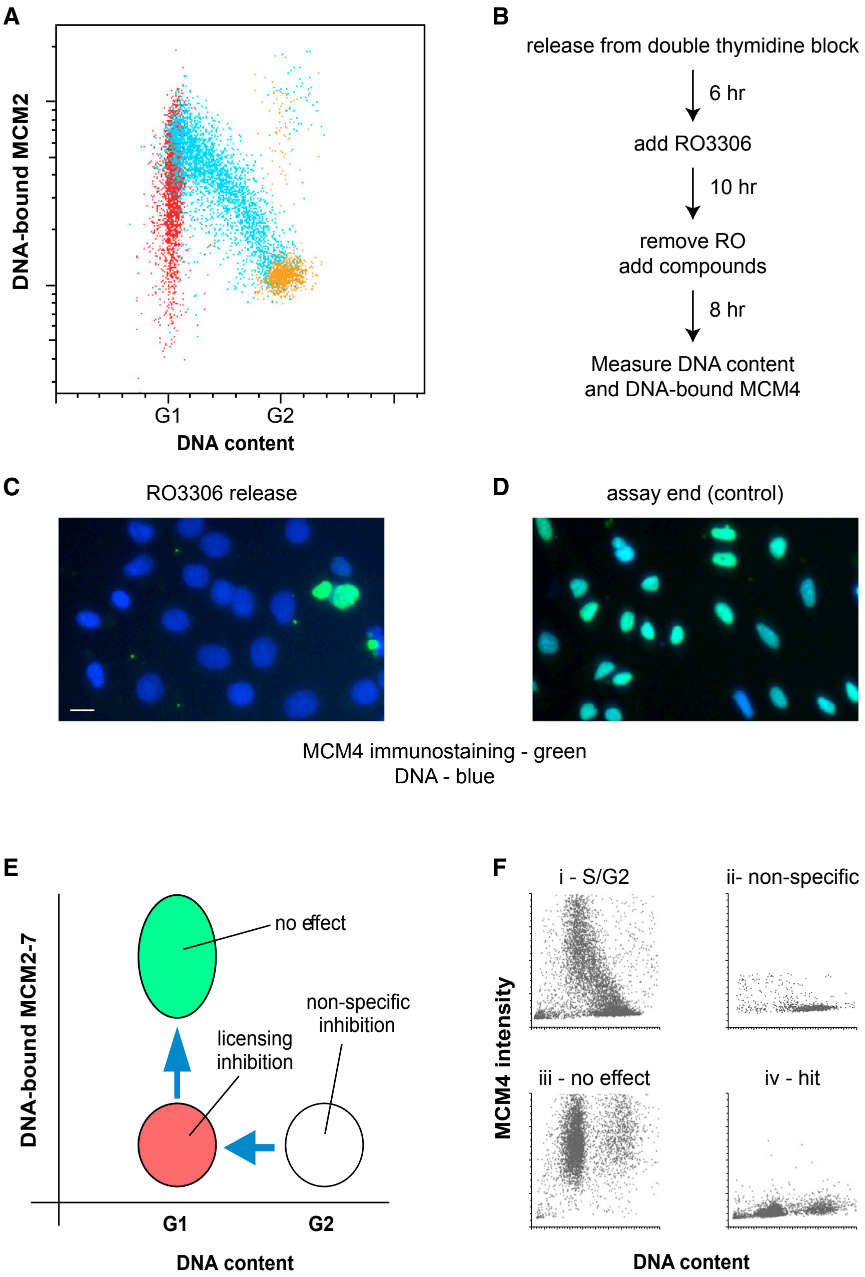


**Figure 1. A Cell-Based Screen for Licensing Inhibitors**

(A) Asynchronous U2OS cells were pulsed with EdU for 30 min, then labeled for EdU incorporation, chromatin-bound MCM2, and total DNA, and analyzed by flow cytometry. For the plot, $G_1$ cells ($G_1$ DNA content, EdU negative) were colored red, S-phase cells (EdU positive) were colored blue, and $G_2$ cells ($G_2$ DNA content, EdU negative) were colored orange, and then plotted for total DNA content and chromatin-bound MCM2.

(B) Outline of the protocol for assaying potential licensing inhibitors.

(C and D) Immunofluorescence images of cells after the RO3306 block (C) or 8 hr later at the end of the assay (D) immunostained for MCM4 (green) and with DAPI for DNA (blue). Scale bar, 10 μm.

(E) Cartoon of possible outcomes of the screen derived from total cellular DNA content and amount of chromatin-bound MCM2–7.

(F) Examples of output from the cell-based screen: (i) late S/$G_2$ enriched starting cells; (ii) non-specific inhibition, showing $G_2$ accumulation; (iii) no inhibition, showing licensed $G_1$ cells; (iv) hit compound showing unlicensed $G_1$ cells.

See also Figures S1 and S6.

p53 and the CDK inhibitors p21$^{Cip1}$ and p27$^{Kip1}$ (Liu et al., 2009; Machida et al., 2005; Nevis et al., 2009; Teer et al., 2006). This reduction in $G_1$ CDK levels prevents the hyperphosphorylation of the pRb transcriptional repressor, thereby blocking the transcriptional program required for cells to enter S phase. This leaves the cells at a stage of $G_1$ where the licensing system is still active.

Importantly, the licensing checkpoint is defective in many cancer cells, perhaps due to the involvement of p53, Rb, and p21$^{Cip1}$, which are mutated or ineffective in many cancers (Shreeram et al., 2002; Feng et al., 2003; Montagnoli et al., 2004; Liu et al., 2009; Nevis et al., 2009; Blow and Gillespie, 2008). This makes the licensing system an attractive anti-cancer target with a potentially high therapeutic index for many different sorts of cancers. Normal cells treated with licensing inhibitors should engage the licensing checkpoint and arrest in $G_1$, capable of completing licensing when the inhibitor is withdrawn; in contrast, cancer cells lacking the licensing checkpoint will enter S phase with an insufficient number of licensed origins, a situation that is almost certainly lethal (Blow and Gillespie, 2008).

In this article we describe a cell-based screen for small-molecule inhibitors of the replication licensing system in human cells. This led to the identification of a related chemical family of 2-arylquinolin-4-amines that we call RL5, which inhibits licensing both in human tissue culture cells and in biochemically tractable *Xenopus* egg extracts. We show that RL5a, the most potent of these compounds identified to date, prevents the tight association of ORC with DNA that is required for replication licensing to occur. ORC binding to DNA requires ATP, but ATP titration shows that RL5a acts non-competitively with respect to ATP.

## RESULTS

### A High-Throughput Cell-Based Screen for Licensing Inhibitors

We have recently devised a 3-dimensional (3D) fluorescence-activated cell sorting (FACS) assay to simultaneously measure the loading of MCM2–7 onto chromatin, 5-ethynyl-2′-deoxyuridine (EdU) incorporation (as a measure of DNA synthesis), and cellular DNA content (Moreno et al., 2016). Figure 1A shows a

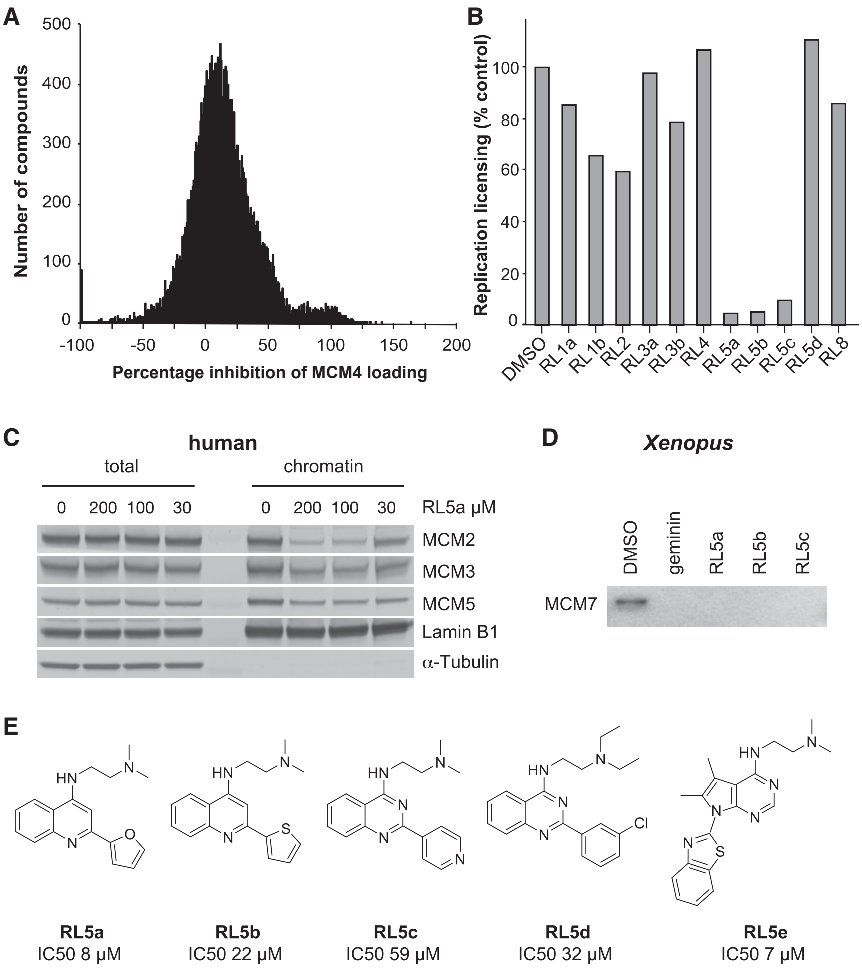


**Figure 2. Licensing Assays in Human and *Xenopus* Systems**

(A) Percentage inhibition of MCM4 loading observed with all 24,000 compounds in the initial screen.

(B) Validated hits from the primary and secondary screen in U2OS cells were assayed for their ability to inhibit an *in vitro* licensing assay in *Xenopus* egg extracts. The degree of licensing is expressed as a percentage of that observed in control "Licensing Factor" extract.

(C) Immunoblot of total and chromatin-bound MCM2, MCM3, and MCM5 in U2OS cells treated with either RL5a or DMSO.

(D) Immunoblot of chromatin-bound MCM7 from *Xenopus* "Licensing Factor" extract treated with DMSO, geminin, RL5a, RL5b, or RL5c.

(E) Structures of RL5a–e.

See also Figures S2 and S6.

2D plot of these data, with chromatin-bound MCM2 on the y axis, DNA content on the x axis, and information from EdU incorporation color coded ($G_1$ red, S phase blue, $G_2$ orange). At cytokinesis, newborn daughter cells have a 2N DNA content with low levels of DNA-bound MCM2. During $G_1$ MCM2 is loaded onto DNA until it reaches a maximum, which likely represents cells satisfying the "licensing checkpoint." MCM2 is progressively displaced as DNA is replicated during S phase, until DNA-bound MCM2 falls to background levels in $G_2$.

We used these changes to design a high-throughput assay for licensing inhibitors (Figure 1B). Human U2OS cells were released into S phase from a double-thymidine block (Figures S1A–S1C) and then treated with RO3306, a CDK1 inhibitor, to reversibly block them in $G_2$ (Figure S1D). RO3306 was removed and cells were seeded into 384-well plates containing test compounds (Figure S1E). Eight hours later, when cells should have passed through mitosis and loaded MCM2–7, cells were fixed and immunostained for DNA-bound MCM4 and also treated with DAPI to stain total DNA (Figure S1F). Microscopic images of each treatment (Figures 1C and 1D) were taken on an InCell 1000 system, which returned values for MCM4 and DNA content for each of the cells identified.

Figure 1E shows a schematic of possible outcomes of this procedure, and Figure 1F shows some example results from the screen. At the time of addition of test compounds, cells had low levels of DNA-bound MCM4 and a $G_2$ DNA content (Figure 1E, white circle). Cells essentially unaffected by test compounds then passed through mitosis to acquire a $G_1$ DNA content and high levels of DNA-bound MCM4 (Figure 1E, green circle; Figure 1Fiii, "no effect"). When exposed to compounds that specifically inhibit licensing, cells passed through mitosis into $G_1$ without acquiring high levels of DNA-bound MCM4 (Figure 1E, red circle; Figure 1Fiv, "hit"). When exposed to compounds that cause non-specific inhibition of essential cellular functions, cells failed to pass through mitosis and maintained a $G_2$ DNA content (Figure 1E, white circle; Figure 1Fii, "non-specific").

The 24,000 small-molecule compound collection held by the Drug Discovery Unit at the University of Dundee was tested at 200 μM. The screen performance indicators were signal to background 2.4 ± 0.53 and *Z*-prime 0.47 ± 0.10. Figure 2A shows the frequency distribution of inhibition of loading of MCM4 that was obtained. There was an approximately normal distribution but with a shoulder between 70% and 130%, indicating the presence of a subpopulation of hits, which do not fit to the expected normal distribution population. Hit compounds are usually defined by a statistical cutoff of mean ± 3 SDs, which in this case are represented by the 349 compounds that gave ≥96.72 percentage inhibition. To minimize the chance of excluding true-positive compounds, we decided to focus on the compounds in the shoulder, and adjusted the initial cutoff value to 70%, resulting in progression of 929 compounds for retesting in duplicate. A total of 280 compounds demonstrated ≥60% inhibition in both replicates (30.1% confirmation rate).

To avoid any confounding effects of RO3306 treatment, we then retested these 280 compounds using a low-throughput screen whereby cells were synchronized by mitotic shake-off prior to exposure with test compounds. This low-throughput

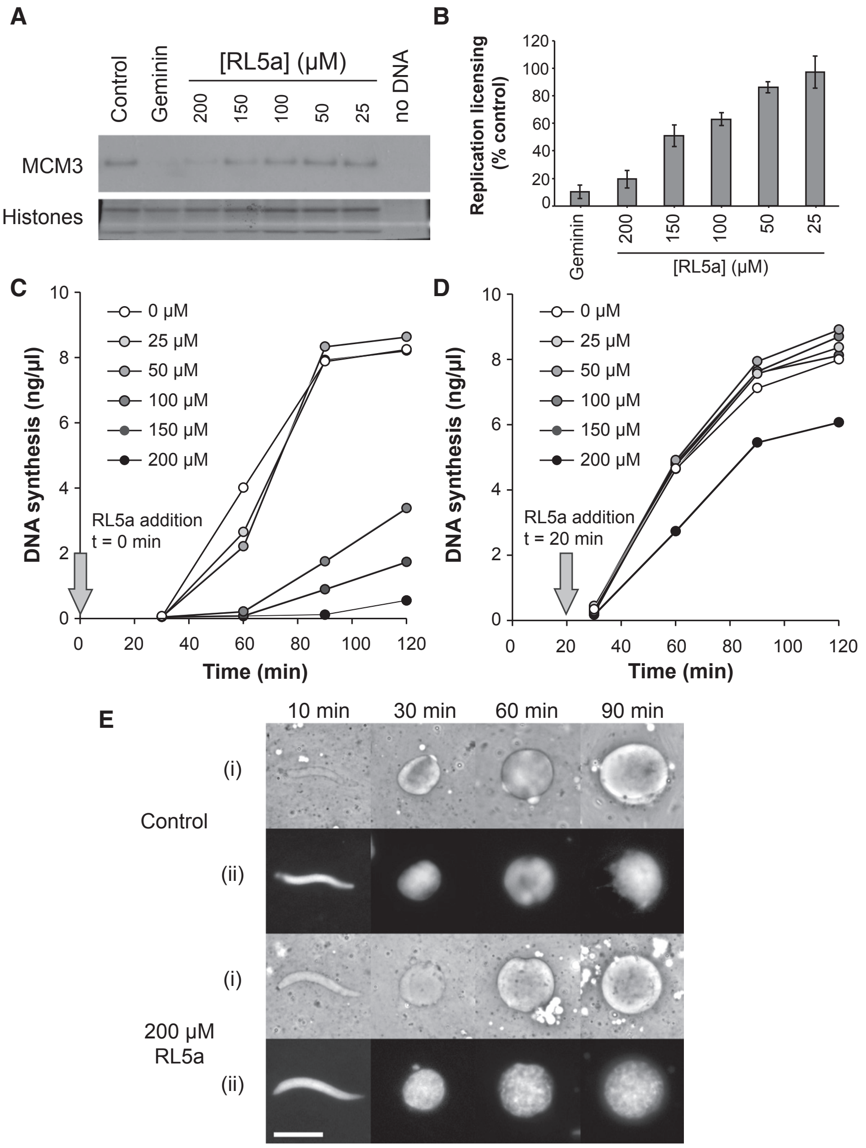


**Figure 3. Activity of RL5a in Whole *Xenopus* Egg Extract**

(A) Sperm nuclei were incubated in whole *Xenopus* egg extract supplemented with 25, 50, 100, 150 or 200 μM RL5a, DMSO, or geminin. After 20 min chromatin was isolated and immunoblotted for MCM3 or stained with Coomassie to show histones.

(B) MCM3 chromatin association in (A) was quantified relative to control (mean ± SEM, n = 4).

(C and D) Sperm nuclei were incubated in whole *Xenopus* egg extract supplemented with [$\alpha$-$^{32}$P] dATP. At either the time of DNA addition (C) or 20 min later (D), extract was additionally supplemented with 0, 25, 50, 100, 150, or 200 μM RL5a in DMSO. At the indicated times, the total amount of DNA synthesized was determined by trichloroacetic acid precipitation and scintillation counting.

(E) Sperm nuclei were incubated in whole egg extract supplemented with DMSO or 200 μM RL5a. At the indicated times nuclear formation was assessed by either (i) phase contrast or (ii) UV microscopy. Scale bar, 20 μm.

See also Figures S3 and S4.

assay also has the advantage of removing cells that suffer non-specific inhibition by test compounds, as only metabolically active cells can re-adhere to the plates. Sixteen compounds produced a reproducible reduction (>60%) of DNA-bound MCM4 in this assay. These 16 compounds fell into eight discrete chemical families, which we named RL1 to RL8. After resynthesis and re-assay, we decided to take 12 of these 16 compounds forward for further study: RL1a, RL1b, RL2, RL3a, RL3b, RL4, RL5a–RL5e, and RL8. Figure S2 shows titration curves for these 12 compounds.

We next aimed to distinguish compounds that directly inhibit replication licensing from compounds that have secondary effects indirectly inhibiting licensing. To do this we used a cell-free system from *Xenopus* (frog) eggs that supports efficient replication licensing *in vitro* (Blow and Laskey, 1988; Blow, 1993; Chong et al., 1995). This system has been reconstituted with purified proteins and is fairly well understood biochemically (Gillespie et al., 2001). The system also shows strong complementarity with the equivalent reaction taking place in mammalian cells, as the proteins required for licensing in the *Xenopus* system (Gillespie et al., 2001) can be substituted by equivalents from mammalian cells (Vashee et al., 2003; Sasaki et al., 2011). In the licensing assay, the selected compounds gave varying degrees of inhibition (Figure 2B); most striking was the behavior of the RL5 family, three members of which (RL5a, b, and c), but not the highly related RL5d, showed very strong inhibition. RL5a, b, c, and d belong to a family of 2-arylquinolin-4-amines, and their chemical structures, together with that of the related RL5e, are shown in Figure 2E. It should be noted that the failure of other compounds in this assay might be due to differences in the exact amino acid sequences of the human and *Xenopus* homologs of the licensing proteins.

We next used chromatin isolation and immunoblotting to directly show that, as expected from our functional assays, RL5 compounds prevented the loading of MCM2–7 proteins onto DNA in human U2OS cells (Figure 2C) and *Xenopus* egg extracts (Figure 2D). Because of its slightly higher potency, we concentrate on the activity of RL5a in the rest of this article (Figures 2B and S2).

We next examined the activity of RL5a in whole *Xenopus* egg extract to determine the potency for inhibition of replication licensing relative to other non-specific effects. RL5a was titrated into interphase egg extract, sperm nuclei were added as template DNA, and the ability for MCM2–7 to be loaded onto DNA was quantified by isolating chromatin and immunoblotting for MCM3 (Figures 3A and 3B). Geminin, a small protein inhibitor of replication licensing (McGarry and Kirschner, 1998;

CellPress

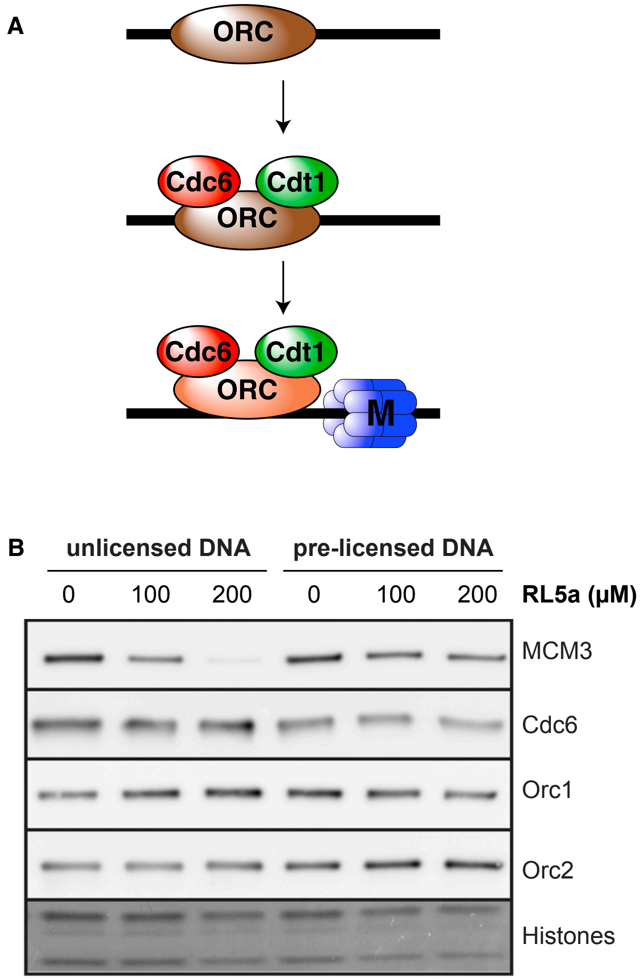


**Figure 4. Effect of RL5a on Unlicensed and Pre-licensed DNA**

(A) Cartoon of the sequential loading of ORC, Cdc6, Cdt1, and MCM2–7 onto origin DNA.

(B) Sperm nuclei were incubated in *Xenopus* egg extract. At the time of DNA addition ("unlicensed DNA") or 15 min later (pre-licensed DNA), extract was optionally supplemented with geminin, DMSO, or the indicated concentrations of RL5a. After a further 20 min, chromatin was isolated and immunoblotted for ORC subunits (Orc1 and Orc2), Cdc6, and MCM3. The bottom of the gel was stained with Coomassie to show histones.

Wohlschlegel et al., 2000; Tada et al., 2001), was used as a control. Figures 3A and 3B show a relatively smooth dose-response curve, with 200 μM RL5a almost completely blocking MCM3 loading. In parallel, we assayed the ability of RL5a to block DNA replication.

There is a complex relationship between the inhibition of MCM loading and inhibition of replication. Previous results in *Xenopus* showed that when licensing was partially inhibited with geminin so that MCM loading was reduced by ~90% bulk DNA replication was only slightly inhibited, but when MCM loading was further reduced the rate of DNA synthesis dropped sharply (Woodward et al., 2006). Figure 3C shows that 25 and 50 μM RL5a had a negligible effect on replication, but 100, 150, and 200 μM showed extensive inhibition. The effect of RL5a shown in Figures 3B and 3C is consistent with RL5a primarily inhibiting replication by inhibition of licensing, but also suggests additional non-specific inhibition of replication.

To obtain an idea of how specific the inhibition of RL5a is to replication licensing, we modified the replication assay: instead of adding RL5a at the start of the incubation, we added DNA to the extract and then added RL5a 20 min later. Within this first 20-min period all origins become licensed in the egg extract, and subsequent inhibition of the licensing system has no effect on DNA replication (Gillespie and Hirano, 2004; Oehlmann et al., 2004; Woodward et al., 2006). However, after 20 min the extract still needs to assemble template DNA into functional interphase nuclei, import nuclear proteins, activate DDK and CDK kinases, initiate replication forks at licensed origins, and perform the elongation stage of DNA replication (Blow and Watson, 1987; Newport, 1987; Sheehan et al., 1988; Blow and Sleeman, 1990; Cox, 1992). Therefore, inhibition of DNA synthesis by RL5a added at 20 min reflects inhibition of these other processes and thus gives an indication of its specificity as a licensing inhibitor. Figure 3D shows that when added 20 min after the template DNA, 200 μM RL5a caused only a modest inhibition of subsequent DNA replication while 150 μM RL5a had no significant effect (Figure 3D). Consistent with this interpretation, Figure 3E shows that even when 200 μM RL5a is added at the start of the incubation, nuclei form normally. Furthermore, when chromatin was isolated from *Xenopus* egg extract at various times and total protein content was analyzed by SDS-PAGE, RL5a caused very little difference in the loading of proteins other than MCM2–7 onto chromatin (Figure S3). We therefore conclude that RL5a has a fair degree of selectivity for inhibiting replication licensing, but also has some inhibitory activity against other cellular activities required for DNA replication. Consistent with this, when 10 μM RL5a was added to U2OS cells following release from a double-thymidine synchronization, it delayed passage through S phase even though no further licensing occurs at this stage of the cell cycle (Figure S4).

## RL5a Prevents ORC Forming a Tight Complex with DNA

The licensing of DNA occurs in a multistep reaction that requires a minimum of four proteins: ORC, Cdc6, Cdt1, and MCM2–7 (Gillespie et al., 2001; Evrin et al., 2009; Remus et al., 2009). The process, which is outlined in Figure 4A, also involves the hydrolysis of ATP and results in double hexamers of MCM2–7 being wrapped around double-stranded DNA (Chong et al., 1995; Gillespie et al., 2001; Evrin et al., 2009; Remus et al., 2009; Gambus et al., 2011). Since this is an energy-dependent reaction, we first asked whether RL5a blocked the loading of MCM2–7 onto DNA or whether it could also promote the unloading of MCM2–7 that was already bound to DNA. When added to *Xenopus* egg extract before template DNA, RL5a efficiently inhibited MCM3 loading onto DNA, consistent with previous assays (Figure 4B, "unlicensed DNA"). However, when MCM2–7 was allowed to load onto DNA and RL5a was added afterward, MCM3 remained bound to DNA (Figure 4B, "pre-licensed DNA"). We conclude that RL5a prevents the loading of MCM2–7 onto DNA but does not significantly promote the unloading of DNA-bound MCM2–7. This is consistent with the experiment shown in Figure 3D, where DNA replication still occurred when RL5a was added to extract 20 min after template DNA.

CellPress

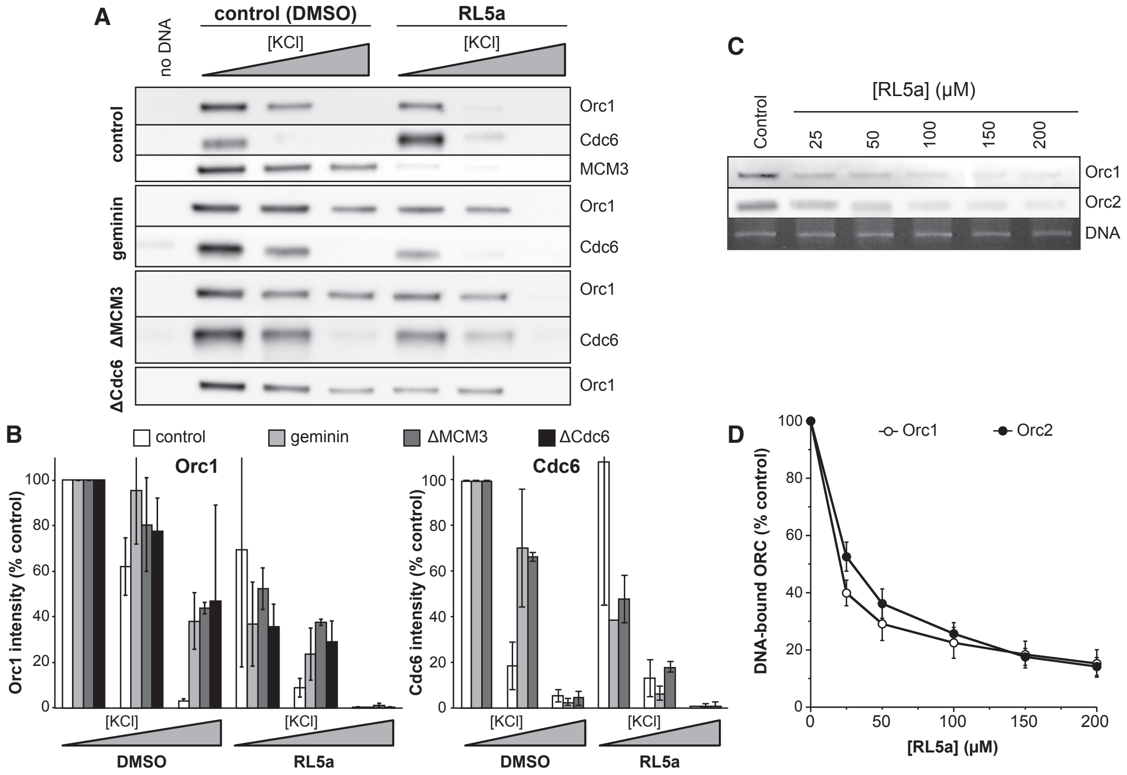


**Figure 5. Effect of RL5a on Licensing-Defective Extracts**

Sperm nuclei were incubated in *Xenopus* egg extract that had optionally been depleted of MCM3 or Cdc6 or which had been supplemented with geminin and which were further supplemented with 200 μM RL5a or DMSO. After 20 min, chromatin was isolated in 50, 100, or 200 mM KCl and then immunoblotted for ORC (Orc1 subunit) or Cdc6.

(A and B) A representative series of blots is shown in (A). Blots from at least three separate experiments were quantified for the amount of chromatin-bound ORC (Orc1 subunit) and Cdc6. The mean signal ± SEM, relative to control chromatin isolated in 50 mM KCl, is plotted in (B).

(C) Plasmid DNA (pET28) was incubated in a partially purified fraction of ORC supplemented with 2.5 mM ATP and either 25, 50, 100, 150, or 200 μM RL5a or DMSO. After 30 min, DNA was isolated in 100 mM KCl and then immunoblotted for Orc1 and Orc2.

(D) Orc1 and Orc2 plasmid DNA association in (C) was quantified relative to recovered DNA and expressed in relation to control (mean ± SEM, n ≥ 5).

See also Figure S5.

We next investigated the stage of the licensing reaction that is inhibited by RL5a. Figure 4B shows that when extract was treated with RL5a, ORC (both the Orc1 and Orc2 subunits) and Cdc6 still bound to DNA. As will become important for the interpretation of subsequent experiments, RL5a did not prevent the association of ORC or Cdc6 with DNA that had already been licensed (Figure 4B, "pre-licensed DNA"). Assays to measure Cdt1 binding are complicated by the relative insolubility of Cdt1 (Tada et al., 2001) and its requirement for ATP in the binding reaction (Evrin et al., 2013; Randell et al., 2006; Remus et al., 2009). We therefore first concentrated on determining any possible effect of RL5a on ORC and Cdc6.

It has previously been shown, in *Xenopus* egg extract, *Saccharomyces cerevisiae*, and *Caenorhabditis elegans* early embryos, that the affinity of ORC and Cdc6 for DNA differs depending on whether or not the DNA is licensed (Rowles et al., 1999; Oehlmann et al., 2004; Tsakraklides and Bell, 2010; Sonneville et al., 2012). Before licensing has occurred, ORC and Cdc6 bind to DNA relatively tightly, but this binding is significantly loosened once origins have been licensed. This behavior is shown in Figure 5, where chromatin was isolated from extracts supplemented with either RL5a or DMSO control and exposed to 50, 100, or 200 mM KCl. Example immunoblots for chromatin-bound ORC (the Orc1 subunit) and Cdc6 are shown in Figure 5A, and the levels of Orc1 and Cdc6 in replicate experiments are plotted in Figure 5B. In control extracts treated with DMSO, where origin licensing does not occur, both ORC and Cdc6 were present on chromatin treated with 50 mM KCl but were removed by higher KCl concentrations: most of the Cdc6 and approximately half the ORC was removed in 100 mM KCl, and neither protein was abundant on chromatin treated with 200 mM KCl. When licensing was blocked, either by the Cdt1 inhibitor geminin or by depletion of MCM3 or Cdc6, the amount of ORC and Cdc6 remaining on chromatin at 100 and 200 mM KCl was significantly increased, indicating their tight binding to DNA before licensing has occurred (Rowles et al., 1999; Oehlmann et al., 2004; Sonneville et al., 2012). Since RL5a blocks licensing, we might expect it to promote the tight binding of ORC and Cdc6 to DNA that is resistant to high salt exposure. However, in extract treated with RL5a, both ORC and Cdc6 were readily removed from DNA by 100 or 200 mM KCl. This indicates that RL5a prevents ORC and

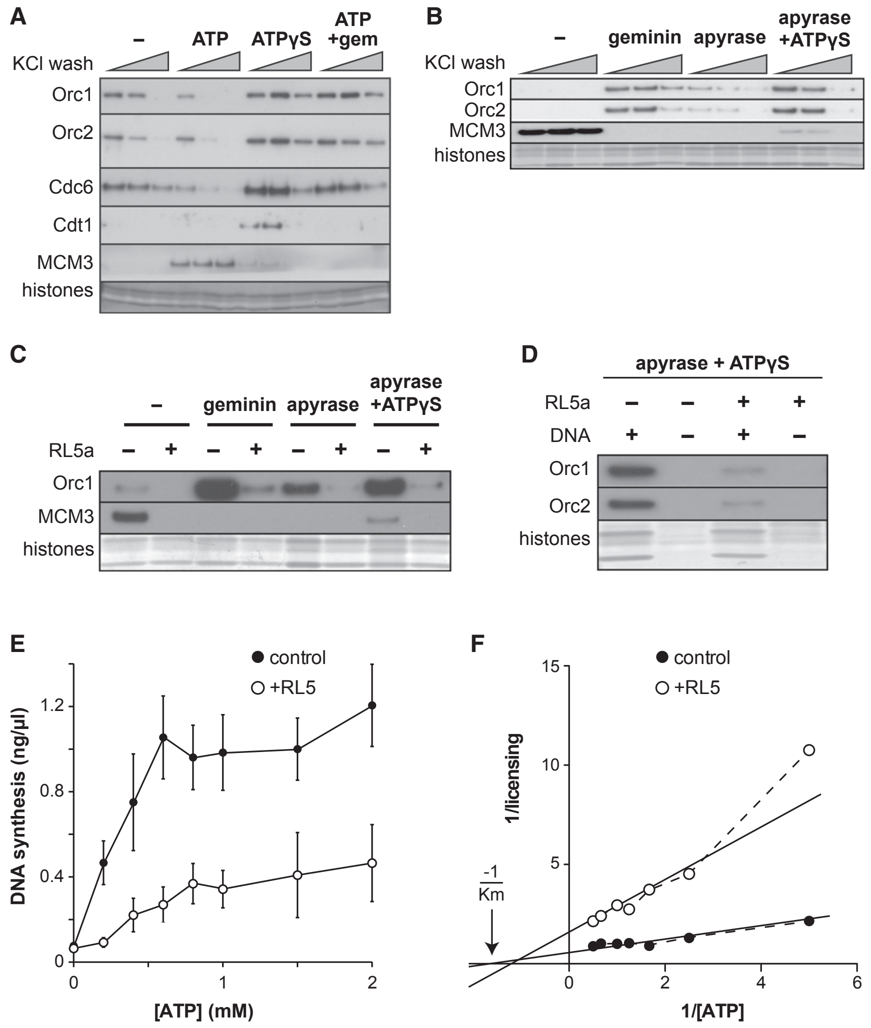


**Figure 6. ATP Requirement for ORC and Cdc6 DNA Binding**

(A) Whole *Xenopus* egg extract was desalted and then supplemented with 2.5 mM ATP or ATP-γ-S plus or minus geminin. Sperm nuclei were incubated in extract for 20 min and isolated in 50, 100, or 200 mM KCl, then immunoblotted for ORC (Orc1 and Orc2 subunits), Cdc6, Cdt1, and MCM3. The bottom of the gel was stained with Coomassie to show histones.

(B) Sperm nuclei were incubated in whole *Xenopus* egg extract that had optionally been treated with geminin, apyrase, or ATP-γ-S. After 20 min, chromatin was isolated in 50, 100, or 200 mM KCl and then immunoblotted for ORC (Orc1 and Orc2 subunits) and MCM3. The bottom of the gel was stained with Coomassie to show histones.

(C and D) Sperm nuclei were incubated in whole *Xenopus* egg extract that had optionally been treated with geminin, apyrase, ATP-γ-S, or RL5a. After 20 min, chromatin was isolated in 100 mM KCl and then immunoblotted for ORC (Orc1 and Orc2 subunits) and MCM3. The bottom of the gel was stained with Coomassie to show histones.

(E and F) *Xenopus* egg extract was depleted of ATP by precipitation with polyethylene glycol, then supplemented with the indicated concentrations of ATP plus or minus 200 μM RL5a. Sperm nuclei were incubated in the reconstituted extract for 15 min, and the degree of licensing obtained was determined by assaying for replication in extract supplemented with geminin. Raw values for the mean ± SEM of three independent experiments (E) and a Lineweaver-Burk plot of the inverse values (F) are shown.

Cdc6 from forming a tight complex with DNA that normally occurs before licensing takes place.

When chromatin was isolated from extract in which licensing had been inhibited with geminin, Orc1, Orc2, and Orc3 were discernible on SDS-PAGE gels stained for total protein (Figure S5). Under these conditions, increasing concentrations of RL5a reduced the chromatin association of these ORC subunits but otherwise had very little effect on the loading of proteins onto unlicensed chromatin. Together with Figure S3, this shows that the two major activities whose chromatin association is limited by RL5a are MCM2–7 and ORC. Since MCM2–7 loading onto DNA (origin licensing) is dependent on ORC, this suggests that RL5a inhibits licensing by inhibiting the association of ORC with DNA.

Since in these experiments we are examining the licensing of DNA that is associated with nucleosomes formed into chromatin, it is possible that RL5a acts on the nucleosomes to inhibit ORC binding (Lipford and Bell, 2001). To test this, we prepared a partially purified fraction of *Xenopus* egg extracts that contains ORC but is not competent to support nucleosome assembly (Gillespie and Blow, 2000; Gillespie et al., 2001). Figure 5C shows that in this partially purified fraction of ORC, RL5a inhibits ORC binding to naked plasmid DNA. Importantly, the extent to which RL5a restricts ORC binding to plasmid DNA (Figure 5D) is similar to that seen on chromatin (Figures 5B and S5) and also matches the inhibition of licensing by RL5a (Figures 3A and 3B). Taken together, these results strongly suggest that RL5a inhibits licensing by directly inhibiting the association of ORC with DNA and that this activity is not mediated by indirect effects on chromatin.

## RL5a Inhibits ORC Allosterically to Mimic a Lack of ATP

ORC and Cdc6 both have ATPase activity, and their binding to DNA is affected by the presence of ATP (Bell and Stillman, 1992; Speck et al., 2005). We therefore investigated how RL5a affected the ATP requirements for the binding of ORC and Cdc6 to DNA. We used several different ways of manipulating ATP content in *Xenopus* egg extracts. In the first approach, we removed nucleotides and other small molecules from the extract using a desalting column (Figure 6A). When template DNA was incubated in desalted extract, ORC (Orc1 and Orc2 subunits) and Cdc6 were observed to bind to the DNA. However, no DNA-bound Cdt1 was observed and licensing (as evidenced by salt-sensitive MCM3 binding) did not occur. Consistent with previous results (Chong et al., 1995; Gillespie et al., 2001), when the desalted extract was supplemented with ATP, template DNA was licensed and was stably loaded with MCM3. As

a consequence of active licensing, the DNA binding of ORC and Cdc6 was weakened. Figure 6A also shows that when extract was supplemented with ATP-γ-S, a non-hydrolyzable form of ATP, no licensing occurred, but a small amount of MCM3 was observed to be associated with DNA that could be removed by high salt. This is consistent with the recruitment of MCM2–7 to ORC and Cdc6 on DNA without the ATP-dependent clamping of the MCM2–7 hexamer around DNA (Evrin et al., 2009; Remus et al., 2009). ATP-γ-S also caused a significant increase in the DNA binding of ORC (Orc1 and Orc2 subunits), Cdc6, and Cdt1. When ATP was added to the extract but licensing was inhibited with geminin, a similar increase in ORC and Cdc6 was observed. These results suggest that the tightly bound form of ORC and Cdc6 that is seen on unlicensed DNA in whole extract (Figure 5) requires unhydrolyzed ATP.

As an alternative method for analyzing the role of ATP in the licensing reaction and its relationship to inhibition by RL5a, we used apyrase, an enzyme that rapidly hydrolyzes ATP to AMP (Seki and Diffley, 2000). Figure 6B shows that extract treated with apyrase did not load MCM3 onto chromatin, and the binding of ORC (Orc1 and Orc2 subunits) to DNA was weak and highly salt sensitive, similar to their behavior in desalted extract. When ATP-γ-S was added to apyrase-treated extract, it caused a significant increase in the binding of ORC to DNA, similar to the effect of adding ATP-γ-S to desalted extract. We then examined the effect of RL5a on the binding of ORC to DNA in extracts lacking hydrolyzable ATP. Figures 6C and 6D show that RL5a significantly weakened the otherwise salt-resistant binding of ORC to DNA under conditions when licensing was blocked by geminin or in the presence of ATP-γ-S. These experiments show that RL5a prevents the tight binding of ORC to DNA that is observed prior to origin licensing and which requires ATP or ATP-γ-S.

We therefore examined whether RL5a exerts its function by competing with ATP for binding to ORC or whether it acts allosterically. To control ATP concentration, diluted and clarified "licensing factor extract" (LFE) was precipitated with 16% polyethylene glycol; these conditions precipitate all the proteins required for origin licensing (ORC, Cdc6, Cdt1, and MCM2–7) and removes the majority of ATP (Chong et al., 1995). Extract was then resuspended in buffer containing 0.2–2 mM ATP and used to perform a functional licensing assay, plus and minus 100 μM RL5a. The raw values are shown in Figure 6E, while Figure 6F shows the data in a double reciprocal (Lineweaver-Burk) plot. In the absence of RL5a, the ATP titration cut the x axis of the double reciprocal plot at approximately −1.65, suggesting that the Michaelis-Menten constant ($K_M$) of the reaction for ATP is ~0.6 mM. In the presence of RL5a, the slope of the double reciprocal plot was steeper, and cut the x axis at about 1.25 (apparent $K_M$ for ATP of ~0.8 mM). The similarity of the x axis intercepts is consistent with RL5a functioning largely as a noncompetitive inhibitor with respect to ATP.

### Effect of RL5a on Non-transformed Cells

The inhibition of replication licensing should allow discrimination between cells that can engage the licensing checkpoint and those that cannot: whereas normal cells will reversibly arrest in $G_1$ following licensing inhibition, cancer cells lacking the checkpoint will enter an abortive S phase. We therefore compared the effect of a range of concentrations of RL5a (1–100 μM) on U2OS cancer cells, which lack the checkpoint, with the effect on checkpoint intact IMR90 primary fibroblasts (Figure 7A). RL5a caused a strong inhibition of U2OS cell growth, with concentrations of 2 μM and higher completely abolishing an increase in cell number. In contrast, primary IMR90 cells were more resistant to RL5a, proliferating to almost normal levels in 1 μM RL5a. This suggests that there is a fundamental difference between the two cell types, which would be consistent with the functioning of the licensing checkpoint in IMR90 but not U2OS cells.

## DISCUSSION

We describe a cell-based assay for small-molecule inhibitors of the replication licensing system. Replication licensing is an attractive anti-cancer target as normal cells possess a "licensing checkpoint" that many cancer cells lack, most likely because it requires the activity of proteins such as p53, Rb, and $p21^{Cip1}$, which are often lost in cancer. Inhibition of licensing should therefore have a high therapeutic index, directly killing cancer cells lacking the checkpoint, while simply delaying normal cells in $G_1$ phase of the cell cycle (Shreeram et al., 2002; Blow and Gillespie, 2008). We screened 24,000 small-molecule compounds for their ability to prevent the loading of MCM4 onto chromatin in human U2OS cells. This revealed 16 compounds in eight discrete chemical families that significantly inhibited the licensing reaction. To determine their mode of action, these compounds were re-assayed in *Xenopus* (frog) egg extracts, which support replication licensing. The RL5 family of 2-arylquinolin-4-amines also had strong inhibitory activity against replication licensing in the *Xenopus* system.

We used our knowledge of the licensing reaction in *Xenopus* egg extract to determine the precise target of RL5a, the most potent of the RL5 compounds. Licensing involves the sequential DNA loading of the pre-replicative complex proteins ORC, Cdc6, and Cdt1, which act together to clamp double hexamers of MCM2–7 around DNA (Chong et al., 1995; Gillespie et al., 2001; Evrin et al., 2009; Remus et al., 2009; Gambus et al., 2011). Once MCM2–7 have been clamped around DNA ORC, Cdc6, and Cdt1 are no longer required to maintain the licensed state (Hua and Newport, 1998; Rowles et al., 1999). It has previously been shown in *Xenopus* egg extract, *S. cerevisiae*, and *C. elegans* early embryos that an initial tight binding of ORC and Cdc6 to DNA is relaxed once licensing has occurred (Rowles et al., 1999; Oehlmann et al., 2004; Tsakraklides and Bell, 2010; Sonneville et al., 2012). This is thought to allow the recycling of ORC, Cdc6, and Cdt1 so that these proteins can each load multiple MCM2–7 double hexamers and thereby license multiple origins.

We show that RL5a inhibits ORC by preventing it forming a tight complex with DNA that is required for origin licensing. When licensing was blocked by other means—by depleting extracts of MCM2–7 or Cdc6, or by addition of the Cdt1 inhibitor geminin—RL5a blocked the formation of a salt-resistant interaction of ORC with DNA. However, RL5a did not inhibit the weak association of ORC that occurs after origins have been licensed. RL5a inhibits the binding of ORC to DNA in both (physiological) chromatin templates and naked DNA. We therefore conclude that RL5a blocks origin licensing by selectively blocking the

formation of a tight ORC-DNA complex, which is a pre-requisite for the licensing reaction to occur (Figure 7B).

ORC is an ATPase and its tight binding to origin DNA depends on ATP (Bell and Stillman, 1992; Klemm et al., 1997; Gillespie et al., 2001; Bowers et al., 2004; Randell et al., 2006; Li and Stillman, 2012). The ATPase activity of Cdc6 and MCM2–7 are also involved in driving the licensing reaction and in recycling ORC and Cdc6 (Zwerschke et al., 1994; Evrin et al., 2013; Frigola et al., 2013; Coster et al., 2014; Evrin et al., 2014; Kang et al., 2014; Chang et al., 2015). We show here that in *Xenopus* egg extract, the formation of a tight interaction between ORC and DNA is dependent on ATP or non-hydrolyzable ATP-$\gamma$-S (Figure 7Bi and Bii). Under normal circumstances ATP hydrolysis then occurs, which is accompanied by the loading of MCM2–7 double hexamers onto DNA and destabilization of the interaction between ORC and DNA (Chang et al., 2015; Coster et al., 2014; Evrin et al., 2013; Frigola et al., 2013; Kang et al., 2014; Bowers et al., 2004; Randell et al., 2006) (Figure 7Biii and Biv). ORC can be kept tightly bound to DNA by preventing ATP hydrolysis (Gillespie et al., 2001; Bowers et al., 2004; Randell et al., 2006; Evrin et al., 2013).

We show that RL5a destabilizes the interaction between ORC and DNA even if licensing is blocked by geminin or by the replacement of ATP with non-hydrolyzable ATP-$\gamma$-S. RL5a therefore makes ORC behave as though it does not have access to ATP and cannot form a tight complex with DNA. We therefore examined whether RL5a may act as a competitive inhibitor for ATP with respect to the licensing reaction. We showed that the licensing reaction in *Xenopus* egg extract has an apparent $K_M$ for ATP of $\sim$0.6 mM. In the presence of RL5a, the apparent $K_M$ for ATP only increased slightly to $\sim$0.8 mM, suggesting that RL5a does not have a strong ability to compete with ATP in this reaction. Instead, these experiments suggest that RL5a acts non-competitively with respect to ATP and can inhibit the activity of ORC allosterically.

The RL5 family of compounds showed reasonable specificity for inhibition of the licensing system. In *Xenopus* egg extracts many nuclear processes occur normally in the presence of concentrations that strongly inhibit licensing. However, at higher concentrations we detected a number of quite severe effects of RL5a that were not mediated by its inhibition of ORC. In U2OS cells, progression through mitosis occurred in the presence of 200 $\mu$M RL5 compounds, although progression through S phase following release from a double-thymidine synchronization was retarded. All members of the RL5 family that had anti-licensing activity also displayed these non-specific effects (data not shown). RL5a does, however, have attractive physical properties from a chemical tool or drug discovery perspective, as it is small (molecular weight 281) and relatively polar (logD = 1.8). Furthermore, it showed selective inhibition of proliferation of U2OS cancer cells when compared with primary IMR90 cells. While providing a valuable tool to study replication licensing, analogs of RL5a will need to more selectively inhibit replication licensing if they are to be therapeutically useful.

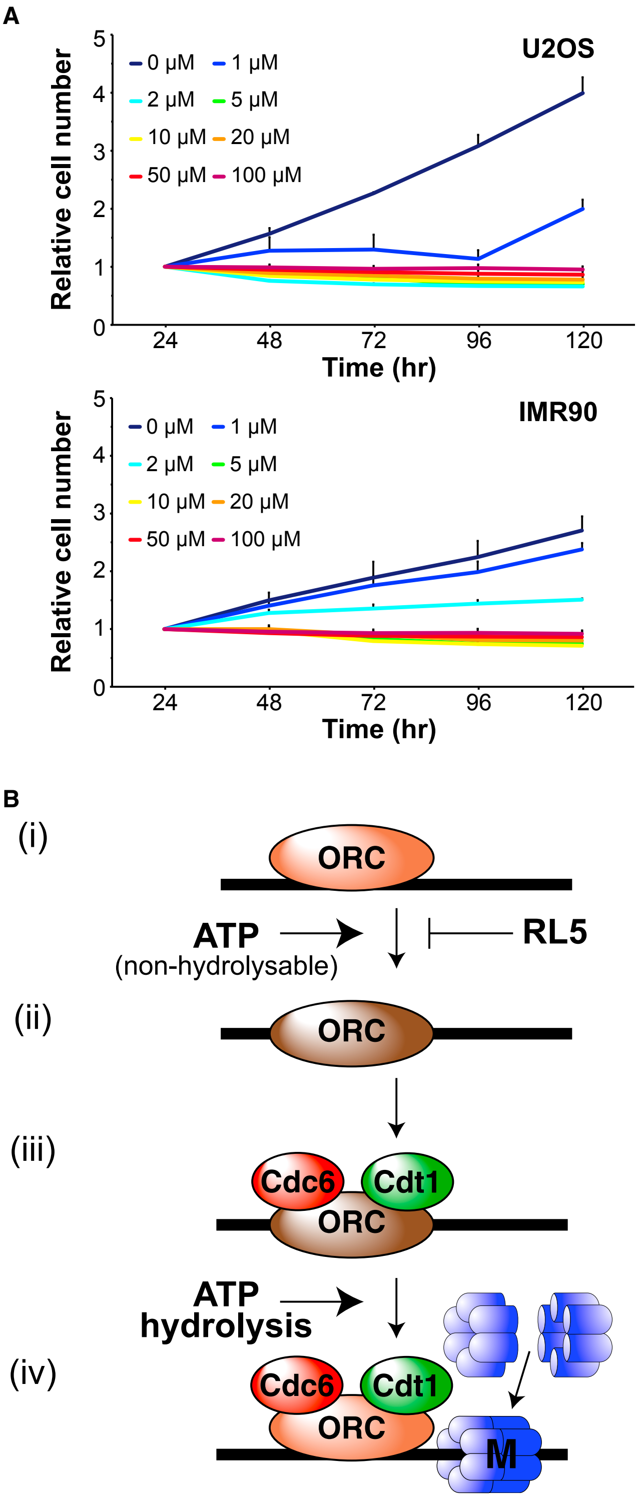


**Figure 7. Depiction of the Licensing Reaction and Its Inhibition by RL5a**

(A) U2OS and IMR90 cells were incubated in 1, 2, 5, 10, 20, 50, or 100 $\mu$M RL5a or DMSO for either 24, 48, 72, 96, or 120 hr and the relative cell number determined. Each data point presented is the mean ± SEM of three biological repeats each formed from three technical repeats.

(B) (i) ORC initially binds weakly to DNA. (ii) In the presence of ATP or ATP-$\gamma$-S, ORC can bind tightly to DNA. This transition is inhibited by RL5a. (iii) Tightly bound ORC further recruits Cdc6 and Cdt1 to DNA. (iv) Double hexamers of MCM2–7 are clamped around DNA as the origin becomes licensed. This requires ATP hydrolysis.

## SIGNIFICANCE

**During S phase of the eukaryotic cell division cycle, pairs of replication forks are initiated at replication origins distributed throughout the genome. Replicative helicase function is provided by activated hexamers of the mini-chromosome maintenance proteins, MCM2–7, which unwind DNA at the head of the replication fork. In late mitosis and $G_1$, future origins of DNA replication are "licensed" for use in the upcoming S phase by being encircled by inactive double hexamers of MCM2–7. To facilitate complete genome duplication during S phase, a "licensing checkpoint" exists to ensure that only cells with a sufficient number of licensed origins progress into S phase. Importantly, the licensing checkpoint is defective in many cancer cells, due to the involvement in checkpoint activity of p53, pRb, and $p21^{Cip1}$, which are mutated or ineffective in many cancers. Whereas normal cells will reversibly arrest in $G_1$ following licensing inhibition, cancer cells lacking the checkpoint will enter an abortive S phase. Inhibition of licensing can therefore selectively kill cancer cells. This makes the licensing system an attractive anti-cancer target with a potentially high therapeutic index for many different sorts of cancers. We describe here a high-throughput cell-based screen for inhibitors of replication licensing with which we identified a family of 2-arylquinolin-4-amines, the most potent of which we call RL5a. The loading of MCM2–7 onto DNA requires the activity of three additional licensing factors: the origin recognition complex (ORC), which binds origin DNA, Cdc6, and Cdt1. We show that RL5a prevents ORC forming a tight complex with DNA that is required for MCM2–7 loading. Furthermore, we show that the proliferation of cells lacking the licensing checkpoint is more sensitive to RL5a than those in which it is intact. This study presents the first description of small-molecule inhibitors of replication licensing that have anti-cancer potential.**

## STAR★METHODS

Detailed methods are provided in the online version of this paper and include the following:

- KEY RESOURCES TABLE
- CONTACT FOR REAGENT AND RESOURCE SHARING
- EXPERIMENTAL MODEL AND SUBJECT DETAILS
  - *Xenopus laevis*
  - Cell Lines
- METHOD DETAILS
  - Flow Cytometry
  - Cell-based Compound Screen
  - Cell Proliferation Assay
  - *Xenopus* Egg Extracts
  - Desalted & Apyrase Treated Extracts
  - Chromatin Isolation
  - Antibodies and Recombinant Protein
  - Polyethylene Glycol Precipitation
  - Licensing Assay
- QUANTIFICATION AND STATISTICAL ANALYSIS

## SUPPLEMENTAL INFORMATION

Supplemental Information includes six figures and can be found with this article online at http://dx.doi.org/10.1016/j.chembiol.2017.06.019.

## AUTHOR CONTRIBUTIONS

N.J.G. established all the cell-based assays, performed the high-throughput screens, analyzed hit profiles, and performed initial analyses using the *Xenopus* cell-free system, including the effect of compounds in immunodepleted extract, and the ATP competition experiment. P.J.G. performed all other mechanistic experiments in *Xenopus* egg extracts. J.T.C. performed the chromatin extraction immunoblot, 2D FACS, and proliferation analyses in cells. E.J.S. and S.P.M. performed the high-throughput screens. A.W. oversaw the high-throughput screens and chemical analysis. E.J.H. performed the 3D cell analysis. J.A.F. and J.J.B. designed the strategy. P.J.G., A.W., and J.J.B. wrote the manuscript.

## ACKNOWLEDGMENTS

This work was supported by CR-UK program grant C303/A14301, Wellcome Trust Investigator award WT096598MA, SULSA project "Inhibitors of replication licensing as novel therapies for cancer," and MRC-DPFS devolved portfolio award MC-G0900864.

Received: May 25, 2016
Revised: June 13, 2017
Accepted: June 30, 2017
Published: August 3, 2017

## REFERENCES

Arias, E.E., and Walter, J.C. (2007). Strength in numbers: preventing rereplication via multiple mechanisms in eukaryotic cells. Genes Dev. *21*, 497–518.

Bell, S.P., and Stillman, B. (1992). ATP-dependent recognition of eukaryotic origins of DNA replication by a multiprotein complex. Nature *357*, 128–134.

Blow, J.J. (1993). Preventing re-replication of DNA in a single cell cycle: evidence for a replication licensing factor. J. Cell Biol. *122*, 993–1002.

Blow, J.J., and Dutta, A. (2005). Preventing re-replication of chromosomal DNA. Nat. Rev. Mol. Cell Biol. *6*, 476–486.

Blow, J.J., and Ge, X.Q. (2009). A model for DNA replication showing how dormant origins safeguard against replication fork failure. EMBO Rep. *10*, 406–412.

Blow, J.J., Ge, X.Q., and Jackson, D.A. (2011). How dormant origins promote complete genome replication. Trends Biochem. Sci. *36*, 405–414.

Blow, J.J., and Gillespie, P.J. (2008). Replication licensing and cancer—a fatal entanglement? Nat. Rev. Cancer *8*, 799–806.

Blow, J.J., and Laskey, R.A. (1988). A role for the nuclear envelope in controlling DNA replication within the cell cycle. Nature *332*, 546–548.

Blow, J.J., and Sleeman, A.M. (1990). Replication of purified DNA in *Xenopus* egg extract is dependent on nuclear assembly. J. Cell Sci. *95*, 383–391.

Blow, J.J., and Watson, J.V. (1987). Nuclei act as independent and integrated units of replication in a *Xenopus* cell-free DNA replication system. EMBO J. *6*, 1997–2002.

Bowers, J.L., Randell, J.C., Chen, S., and Bell, S.P. (2004). ATP hydrolysis by ORC catalyzes reiterative Mcm2-7 assembly at a defined origin of replication. Mol. Cell *16*, 967–978.

Chang, F., Riera, A., Evrin, C., Sun, J., Li, H., Speck, C., and Weinreich, M. (2015). Cdc6 ATPase activity disengages Cdc6 from the pre-replicative complex to promote DNA replication. Elife *4*, e05795.

Chong, J.P., Mahbubani, H.M., Khoo, C.Y., and Blow, J.J. (1995). Purification of an MCM-containing complex as a component of the DNA replication licensing system. Nature *375*, 418–421.

Chong, J.P., Thommes, P., Rowles, A., Mahbubani, H.M., and Blow, J.J. (1997). Characterization of the *Xenopus* replication licensing system. Methods Enzymol. *283*, 549–564.

Coster, G., Frigola, J., Beuron, F., Morris, E.P., and Diffley, J.F. (2014). Origin licensing requires ATP binding and hydrolysis by the MCM replicative helicase. Mol. Cell *55*, 666–677.

Cox, L.S. (1992). DNA replication in cell-free extracts from *Xenopus* eggs is prevented by disrupting nuclear envelope function. J. Cell Sci. *101*, 43–53.

Evrin, C., Clarke, P., Zech, J., Lurz, R., Sun, J., Uhle, S., Li, H., Stillman, B., and Speck, C. (2009). A double-hexameric MCM2-7 complex is loaded onto origin DNA during licensing of eukaryotic DNA replication. Proc. Natl. Acad. Sci. USA *106*, 20240–20245.

Evrin, C., Fernandez-Cid, A., Riera, A., Zech, J., Clarke, P., Herrera, M.C., Tognetti, S., Lurz, R., and Speck, C. (2014). The ORC/Cdc6/MCM2-7 complex facilitates MCM2-7 dimerization during prereplicative complex formation. Nucleic Acids Res. *42*, 2257–2269.

Evrin, C., Fernandez-Cid, A., Zech, J., Herrera, M.C., Riera, A., Clarke, P., Brill, S., Lurz, R., and Speck, C. (2013). In the absence of ATPase activity, pre-RC formation is blocked prior to MCM2-7 hexamer dimerization. Nucleic Acids Res. *41*, 3162–3172.

Feng, D., Tu, Z., Wu, W., and Liang, C. (2003). Inhibiting the expression of DNA replication-initiation proteins induces apoptosis in human cancer cells. Cancer Res. *63*, 7356–7364.

Ferenbach, A., Li, A., Brito-Martins, M., and Blow, J.J. (2005). Functional domains of the *Xenopus* replication licensing factor Cdt1. Nucleic Acids Res. *33*, 316–324.

Frigola, J., Remus, D., Mehanna, A., and Diffley, J.F. (2013). ATPase-dependent quality control of DNA replication origin licensing. Nature *495*, 339–343.

Gambus, A., Khoudoli, G.A., Jones, R.C., and Blow, J.J. (2011). MCM2-7 form double hexamers at licensed origins in *Xenopus* egg extract. J. Biol. Chem. *286*, 11855–11864.

Ge, X.Q., and Blow, J.J. (2010). Chk1 inhibits replication factory activation but allows dormant origin firing in existing factories. J. Cell Biol. *191*, 1285–1297.

Ge, X.Q., Jackson, D.A., and Blow, J.J. (2007). Dormant origins licensed by excess Mcm2 7 are required for human cells to survive replicative stress. Genes Dev. *21*, 3331–3341.

Gillespie, P.J., and Blow, J.J. (2000). Nucleoplasmin-mediated chromatin remodelling is required for *Xenopus* sperm nuclei to become licensed for DNA replication. Nucleic Acids Res. *28*, 472–480.

Gillespie, P.J., Gambus, A., and Blow, J.J. (2012). Preparation and use of *Xenopus* egg extracts to study DNA replication and chromatin associated proteins. Methods *57*, 203–213.

Gillespie, P.J., and Hirano, T. (2004). Scc2 couples replication licensing to sister chromatid cohesion in *Xenopus* egg extracts. Curr. Biol. *14*, 1598–1603.

Gillespie, P.J., Li, A., and Blow, J.J. (2001). Reconstitution of licensed replication origins on *Xenopus* sperm nuclei using purified proteins. BMC Biochem. *2*, 15.

Gillespie, P.J., Neusiedler, J., Creavin, K., Chadha, G.S., and Blow, J.J. (2016). Cell cycle synchronization in *Xenopus* egg extracts. Methods Mol. Biol. *1342*, 101–147.

Harvey, K.J., and Newport, J. (2003). CpG methylation of DNA restricts prereplication complex assembly in *Xenopus* egg extracts. Mol. Cell Biol. *23*, 6769–6779.

Hua, X.H., and Newport, J. (1998). Identification of a preinitiation step in DNA replication that is independent of origin recognition complex and cdc6, but dependent on cdk2. J. Cell Biol. *140*, 271–281.

Ibarra, A., Schwob, E., and Mendez, J. (2008). Excess MCM proteins protect human cells from replicative stress by licensing backup origins of replication. Proc. Natl. Acad. Sci. USA *105*, 8956–8961.

Ilves, I., Petojevic, T., Pesavento, J.J., and Botchan, M.R. (2010). Activation of the MCM2-7 helicase by association with Cdc45 and GINS proteins. Mol. Cell *37*, 247–258.

Kang, S., Warner, M.D., and Bell, S.P. (2014). Multiple functions for Mcm2-7 ATPase motifs during replication initiation. Mol. Cell *55*, 655–665.

Klemm, R.D., Austin, R.J., and Bell, S.P. (1997). Coordinate binding of ATP and origin DNA regulates the ATPase activity of the origin recognition complex. Cell *88*, 493–502.

Lambert, S., and Carr, A.M. (2005). Checkpoint responses to replication fork barriers. Biochimie *87*, 591–602.

Li, H., and Stillman, B. (2012). The origin recognition complex: a biochemical and structural view. Subcell Biochem. *62*, 37–58.

Lipford, J.R., and Bell, S.P. (2001). Nucleosomes positioned by ORC facilitate the initiation of DNA replication. Mol. Cell *7*, 21–30.

Liu, P., Slater, D.M., Lenburg, M., Nevis, K., Cook, J.G., and Vaziri, C. (2009). Replication licensing promotes cyclin D1 expression and G1 progression in untransformed human cells. Cell Cycle *8*, 125–136.

Machida, Y.J., Teer, J.K., and Dutta, A. (2005). Acute reduction of an origin recognition complex (ORC) subunit in human cells reveals a requirement of ORC for Cdk2 activation. J. Biol. Chem. *280*, 27624–27630.

McGarry, T.J., and Kirschner, M.W. (1998). Geminin, an inhibitor of DNA replication, is degraded during mitosis. Cell *93*, 1043–1053.

Montagnoli, A., Tenca, P., Sola, F., Carpani, D., Brotherton, D., Albanese, C., and Santocanale, C. (2004). Cdc7 inhibition reveals a p53-dependent replication checkpoint that is defective in cancer cells. Cancer Res. *64*, 7110–7116.

Moreno, A., Carrington, J.T., Albergante, L., Al Mamun, M., Haagensen, E.J., Komseli, E.S., Gorgoulis, V.G., Newman, T.J., and Blow, J.J. (2016). Unreplicated DNA remaining from unperturbed S phases passes through mitosis for resolution in daughter cells. Proc. Natl. Acad. Sci. USA *113*, E5757–E5764.

Moyer, S.E., Lewis, P.W., and Botchan, M.R. (2006). Isolation of the Cdc45/Mcm2-7/GINS (CMG) complex, a candidate for the eukaryotic DNA replication fork helicase. Proc. Natl. Acad. Sci. USA *103*, 10236–10241.

Nevis, K.R., Cordeiro-Stone, M., and Cook, J.G. (2009). Origin licensing and p53 status regulate Cdk2 activity during G(1). Cell Cycle *8*, 1952–1963.

Newport, J. (1987). Nuclear reconstitution in vitro: stages of assembly around protein-free DNA. Cell *48*, 205–217.

Oehlmann, M., Score, A.J., and Blow, J.J. (2004). The role of Cdc6 in ensuring complete genome licensing and S phase checkpoint activation. J. Cell Biol. *165*, 181–190.

Prokhorova, T.A., and Blow, J.J. (2000). Sequential MCM/P1 subcomplex assembly is required to form a heterohexamer with replication licensing activity. J. Biol. Chem. *275*, 2491–2498.

Randell, J.C., Bowers, J.L., Rodriguez, H.K., and Bell, S.P. (2006). Sequential ATP hydrolysis by Cdc6 and ORC directs loading of the Mcm2-7 helicase. Mol. Cell *21*, 29–39.

Remus, D., Beuron, F., Tolun, G., Griffith, J.D., Morris, E.P., and Diffley, J.F. (2009). Concerted loading of Mcm2-7 double hexamers around DNA during DNA replication origin licensing. Cell *139*, 719–730.

Rowles, A., Chong, J.P., Brown, L., Howell, M., Evan, G.I., and Blow, J.J. (1996). Interaction between the origin recognition complex and the replication licensing system in *Xenopus*. Cell *87*, 287–296.

Rowles, A., Tada, S., and Blow, J.J. (1999). Changes in association of the *Xenopus* origin recognition complex with chromatin on licensing of replication origins. J. Cell Sci. *112*, 2011–2018.

Sasaki, T., Li, A., Gillespie, P.J., Blow, J.J., and Gilbert, D.M. (2011). Evidence for a mammalian late-G1 phase inhibitor of replication licensing distinct from geminin or Cdk activity. Nucleus *2*, 455–464.

Seki, T., and Diffley, J.F. (2000). Stepwise assembly of initiation proteins at budding yeast replication origins in vitro. Proc. Natl. Acad. Sci. USA *97*, 14115–14120.

Sheehan, M.A., Mills, A.D., Sleeman, A.M., Laskey, R.A., and Blow, J.J. (1988). Steps in the assembly of replication-competent nuclei in a cell-free system from *Xenopus* eggs. J. Cell Biol. *106*, 1–12.

Shreeram, S., Sparks, A., Lane, D.P., and Blow, J.J. (2002). Cell type-specific responses of human cells to inhibition of replication licensing. Oncogene *21*, 6624–6632.

Sonneville, R., Querenet, M., Craig, A., Gartner, A., and Blow, J.J. (2012). The dynamics of replication licensing in live *Caenorhabditis elegans* embryos. J. Cell Biol. *196*, 233–246.

Speck, C., Chen, Z., Li, H., and Stillman, B. (2005). ATPase-dependent cooperative binding of ORC and Cdc6 to origin DNA. Nat. Struct. Mol. Biol. *12*, 965–971.

Tada, S., Li, A., Maiorano, D., Mechali, M., and Blow, J.J. (2001). Repression of origin assembly in metaphase depends on inhibition of RLF-B/Cdt1 by geminin. Nat. Cell Biol. *3*, 107–113.

Teer, J.K., Machida, Y.J., Labit, H., Novac, O., Hyrien, O., Marheineke, K., Zannis-Hadjopoulos, M., and Dutta, A. (2006). Proliferating human cells hypomorphic for origin recognition complex 2 and pre-replicative complex formation have a defect in p53 activation and Cdk2 kinase activation. J. Biol. Chem. *281*, 6253–6260.

Tsakraklides, V., and Bell, S.P. (2010). Dynamics of pre-replicative complex assembly. J. Biol. Chem. *285*, 9437–9443.

Vashee, S., Cvetic, C., Lu, W., Simancek, P., Kelly, T.J., and Walter, J.C. (2003). Sequence-independent DNA binding and replication initiation by the human origin recognition complex. Genes Dev. *17*, 1894–1908.

Wohlschlegel, J.A., Dwyer, B.T., Dhar, S.K., Cvetic, C., Walter, J.C., and Dutta, A. (2000). Inhibition of eukaryotic DNA replication by geminin binding to Cdt1. Science *290*, 2309–2312.

Woodward, A.M., Gohler, T., Luciani, M.G., Oehlmann, M., Ge, X., Gartner, A., Jackson, D.A., and Blow, J.J. (2006). Excess Mcm2-7 license dormant origins of replication that can be used under conditions of replicative stress. J. Cell Biol. *173*, 673–683.

Zwerschke, W., Rottjakob, H.W., and Kuntzel, H. (1994). The Saccharomyces cerevisiae CDC6 gene is transcribed at late mitosis and encodes a ATP/GTPase controlling S phase initiation. J. Biol. Chem. *269*, 23351–23356.

# STAR★METHODS

## KEY RESOURCES TABLE

| REAGENT or RESOURCE | SOURCE | IDENTIFIER |
|---|---|---|
| Antibodies | | |
| Alexa Fluor 488 goat anti-mouse antibody | Invitrogen | A11029; RRID: AB_138404 |
| Alexa Fluor 488 goat anti-rabbit antibody | Invitrogen | A11034; RRID: AB_2576217 |
| Mcm2 (BM28) primary antibody | BD Biosciences | 610701; RRID: AB_398024 |
| Mcm3 primary antibody | N/A | Prokhorova and Blow, 2000 |
| Mcm4 primary antibody | Santa Cruz | Sc22779; RRID: AB_2142394 |
| Mcm5 primary antibody | Santa Cruz | Sc136366; RRID: AB_10647089 |
| Mcm7 primary antibody | N/A | Prokhorova and Blow, 2000 |
| Orc1 primary antibody | N/A | Rowles et al., 1996 |
| Orc2 primary antibody | N/A | Oehlmann et al., 2004 |
| Cdc6 primary antibody | N/A | Oehlmann et al., 2004 |
| Cdt1 primary antibody | N/A | Tada et al., 2001 |
| Lamin B1 primary antibody | Abcam | 16048; RRID: AB_443298 |
| $\alpha$-Tubulin primary antibody | Sigma-Aldrich | T6199; RRID: AB_477583 |
| Chemicals, Peptides, and Recombinant Proteins | | |
| Thymidine | Sigma | T1895 |
| RO3306 | Alexis | ALX-270-463 |
| RL5a | Maybridge | 198-004-017 |
| RL5b | Maybridge | 198-004-023 |
| RL5c | Maybridge | 198-004-021 |
| Apyrase | Sigma | A6410 |
| Critical Commercial Assays | | |
| Click-iT Plus EdU Alexa Fluor 647 Flow Cytometry Assay Kit | Invitrogen | C10634 |
| PrestoBlue Cell Viability Reagent | Invitrogen | A13262 |
| Experimental Models: Cell Lines | | |
| U2OS cells | ATCC | HTB-96, Lot 7658494 |
| IMR90 cells | ATCC | CCL-186, Lot 62162583 |
| Experimental Models: Organisms/Strains | | |
| Wild type *Xenopus laevis* (male and female) | University of Plymouth, UK | N/A |

## CONTACT FOR REAGENT AND RESOURCE SHARING

Requests for resources, reagents and further information should be directed to and will be fulfilled by, the Lead Contact, J. Julian Blow (j.j.blow@dundee.ac.uk).

## EXPERIMENTAL MODEL AND SUBJECT DETAILS

### *Xenopus laevis*

Wild type, sexually mature ($\geq$ 1 year old) female and male South African Clawed frogs (*Xenopus laevis*) born and reared in the UK (University of Plymouth) were used in this study for the production of unfertilised eggs (from which extracts were prepared) and sperm, respectively. Frogs were maintained at 19°C in particulate filtered, dechlorinated water, at a density of $\leq$15 animals per 60 L tank, in a purpose built “aquacentre” and were maintained by a professional staff at the University of Dundee adhering to Home Office (UK Government) animal husbandry guidelines; the animals have access to a Home Office (UK Government) approved veterinary surgeon. The frogs were fed a vegetable and fish based diet (Aquatic Diets 3, Mazuri Zoo Foods) 2-3 times per week, as required.

## Cell Lines

U2OS cells (Female; ATCC, Cat. No. HTB-96, Lot 7658494) and IMR90 cells (Female; ATCC, Cat. No. CCL-186, Lot 62162583) were grown in DMEM (Invitrogen, Cat No.12491-023) supplemented with 10% FBS (Invitrogen) plus 100U/ml penicillin, 100 $\mu$g/ml streptomycin (Invitrogen, Cat. No.15070-063) at 37°C with 5% $CO_2$.

# METHOD DETAILS

## Flow Cytometry

3D Flow Cytometry was performed as described (Moreno et al., 2016). Cells were treated with 40 $\mu$M EdU (Invitrogen) for 30 min prior to trypsinization and collection. Cells were pre-extracted with CSK buffer (10mM HEPES pH 7.4, 300 mM Sucrose, 100 mM NaCl, 3 mM $MgCl_2$, 0.5% Triton-X-100) for 10 min on ice and then fixed in 2% formaldehyde for 15 min. Cells were permeabilised in ice-cold 70% ethanol for 10 min and incubated for 1 hr with anti-BM28 (MCM2) primary antibody (1:500) (BD Biosciences, Cat. No. 610701). After staining with Alexa Fluor 488 goat anti-mouse antibody (Invitrogen, Cat. No. A11029) cells were washed and the Click-it EdU reaction (Invitrogen, Cat. No. C10634) was performed for 30 min. Finally, cells were treated with propidium Iodide (PI) solution (50 $\mu$g/ml PI, 50 $\mu$g/ml RNaseA, 0.1% Triton-X-100) and transferred to FACS tubes for analysis. Cells analysed for their DNA content only were fixed and treated with PI as above. All samples were acquired using a BD FACSCanto and results analysed using FlowJo software.

## Cell-based Compound Screen

U2OS cells were synchronized at the $G_1$/S border using a double thymidine block (1 mM thymidine; Sigma, Cat. No. T1895; 16 hr for the first block followed by release for 12 hr and a second block for 18 hr); cells were then seeded onto 384-well plates in the absence of thymidine and 6 hr later were blocked at the G2/M border by addition of 9 $\mu$M RO3306 (Alexis, Cat. No. ALX-270-463, stock made up at 9 mM in dimethyl sulphoxide). After a further 10 hr, cells were released from RO3306 inhibition, washed and then compound added. After incubation for 8 hr the plates were removed to a Janus Automated Workstation where the wells were washed 3 times in PBS. The cells were then extracted with the addition of ice-cold CSK buffer containing 0.3% Triton-X100 (CSK: 10 mM Hepes-KOH pH 7.4, 300 mM sucrose, 100 mM NaCl, 3 mM $MgCl_2$) for 10 min at RT to remove non-chromatin bound Mcms. Wells were then washed once with PBS 0.1% Triton-X100 and then fixed by the addition of -20°C methanol for 5 min. Cells were then washed twice with PBS 0.1% Triton, once with PBS and then were either processed immediately for staining or stored in PBS overnight at 4°C. Plates were then probed with 1: 50 Mcm4 primary antibody (Santa Cruz, Cat. No. Sc22779) followed by 1:300 Alexa Fluor 488 goat anti-rabbit antibody (Invitrogen, Cat. No. A11034) and DAPI (Sigma, Cat. No. D9564).

Microscopic images (6 fields / well) of each treatment were taken on an InCell 1000 system (GE healthcare) using a 20x objective. ActivityBase from ID Business Solutions was used for the data processing and analysis, returning values for Mcm4 and DNA content for each of the cells identified. Figure S6 details the plate maps used for the assay. A Quality Control plate was included for every three assay plates (Figure S6), where columns 1-12 were low control wells (+ R03306 + 1% DMSO) and columns 13-24 were high control well (+Media + 1% DMSO). The performance of the assay on each screening plate was evaluated using internal controls. The criteria for data acceptance of an assay plate were signal to background (mean Hi Control) / (mean Lo Control) >1.5; Z-prime (1 - (3 x Lo Control Standard Deviation + 3 x SD Hi Control Standard Deviation) / (mean Hi Control - mean Lo Control)) >0.3. The statistics from all assay plates and Quality Control plates were analysed. The screen performance indicators were: signal to background 2.4±0.53 and Z-prime 0.47±0.10.

For the secondary screen, mitotic cells were acquired by shake-off from asynchronous cell cultures, spun and re-plated into the test compounds for 2 hr before being processed as above. RL5a, RL5b and RL5c were purchased from Maybridge with product codes 198-004-017, 198-004-023 and 198-004-021 respectively.

## Cell Proliferation Assay

U2OS and IMR90 cells were grown in complete DMEM, as described. Cells, seeded in 96-well plates, were given 24 hr to adhere prior to the addition of RL5a. At the indicated times, 5% (v/v) PrestoBlue Cell Viability Reagent (Invitrogen, Cat. No. A13262) was added to each well and incubated for 1 hr. Fluorescence was quantified using a FLUOstar Omega plate reader (BMG Labtech) and measurements taken at 544 nm excitation and 590 nm emission. The raw data was background corrected using a complete/supplemented DMEM, DMSO and PrestoBlue blank well and normalised against cell number after 24 hr. Each datapoint presented is the mean of 3 biological repeats each formed from 3 technical repeats.

## *Xenopus* Egg Extracts

Metaphase-arrested *Xenopus laevis* egg extract and demembranated *Xenopus* sperm nuclei were prepared as described (Gillespie et al., 2012, 2016).

Female frogs were primed with 150 units of Folligon (Pregnant Mare Serum Gonadotrophin) 3 days before the eggs were required to increase the number of stage 6 mature oocytes and 2 days later, were injected with 500 units Chorulon (Chorionic Gonadotrophin) to induce ovulation. Frogs were placed in individual laying tanks at 18-21°C in 2 l 1x MMR egg laying buffer, prepared from a 10x stock (1 M NaCl, 20 mM KCl, 10 mM $MgCl_2$, 20 mM $CaCl_2$,1 mM EDTA, 50 mM HEPES-NaOH, pH 7.8). The following morning, eggs were collected and rinsed in 1x MMR to remove any non-egg debris. Washed eggs were dejellied in 2% w/v cysteine (pH 7.8), washed in

XBE2 (1x XB salts, 1.71% w:v sucrose, 5 mM K-EGTA, 10 mM HEPES-KOH, pH 7.7; 10x XB salts: 2 M KCl, 40 mM $MgCl_2$, 2 mM $CaCl_2$) and then into XBE2 containing 10 μg/ml leupeptin, pepstatin and aprotinin. Dejellied and washed eggs were centrifuged in 14 ml tubes, containing 1ml XBE2 plus protease inhibitors containing 100 μg/ml cytochalasin D, at 1400 x g in a swinging bucket rotor for 1 min at 16°C to pack the eggs, after which excess buffer and dead eggs were removed. Packed eggs were crushed by centrifugation at 16,000 x g in a swinging bucket rotor for 10 min at 16°C. The dirty brown cytoplasmic layer was collected using a 20G needle and a 1 ml syringe via side puncture. From this point onwards the extract was kept on ice. The crude extract was supplemented with cytochalasin D, leupeptin, pepstatin and aprotinin all to a final concentration of 10 μg/ml, 1:80 dilution of energy regenerator (1 M phosphocreatine disodium salt, 600 μg/ml creatine phosphokinase in 10 mM HEPES-KOH pH 7.6) and 15% v:v LFB1/50 (10% w:v sucrose, 50 mM KCl, 2 mM $MgCl_2$, 1 mM EGTA, 2 mM DTT, 20 mM $K_2HPO4/KH_2PO4$ pH 8.0, 40 mM HEPES-KOH, pH 8.0). The extract was clarified by centrifugation at 84,000 x g in a pre-cooled SW55 rotor swinging bucket rotor at 4°C for 20 min. The golden cytoplasmic layer was recovered, supplemented with glycerol to 2% v/v and frozen in aliquots in liquid nitrogen and stored at -80°C until required.

Sperm was recovered from testes isolated from male frogs post mortem following a lethal dose of anaesthetic (0.2% w:v Tricaine mesylate MS222, ~0.5% w:v NaHCO3, to pH 7.5). Isolated testes were washed carefully to avoid bursting in EB (50 mM KCl, 5 mM $MgCl_2$, 2 mM dithiothreitol or β-mercaptoethanol, 50 mM HEPES-KOH, pH 7.6), prior to being finely chopped with a clean razor blade in fresh EB. Recovered lysate was filtered through a 25 μm nylon membrane to remove particulate matter. Filtered sperm was centrifuged at 2,000 x g at 4°C for 5 min; selective resuspension of the sperm pellet allowed separation of the sperm from contaminating erythrocytes; the resuspended sperm was respun and the pellet resuspended in 0.5 ml SuNaSp (0.25 M sucrose, 75 mM NaCl, 0.5 mM spermidine, 0.15 mM spermine, 15 mM HEPES-KOH, pH 7.6) per testis. The sperm was demembranated with the addition of 25 μl per testis lysolecithin (5 mg/ml, in $H_2O$) for 10 min at room temperature. Demembranated sperm were respun and resuspended in SuNaSp plus 3% w/v BSA to quench the demembranation reaction. Quenched sperm were respun and resuspended in EB plus 30% glycerol per testis, counted using a haemocytometer and stored at -80°C.

Extracts were supplemented with 250 μg/ml cycloheximide, 25 mM phosphocreatine and 15 μg/ml creatine phosphokinase and incubated with 0.3 mM $CaCl_2$ for 15 minutes to trigger release from metaphase arrest. For DNA synthesis reactions, demembranated *Xenopus* sperm nuclei were incubated at 6-10 ng DNA/μl in extract. DNA synthesis was assayed by measuring incorporation of [α-$^{32}$P]dATP into acid-insoluble material followed by scintillation counting, as described (Gillespie et al., 2012, 2016). Extract was supplemented with 50 nCi/μl [α-$^{32}$P]dATP from a high activity 10 mCi/ml stock. At the appropriate times 10 μl aliquots were stopped by the addition of 160 μl Stop-C (0.5% w:v SDS, 5 mM EDTA, 20 mM Tris HCl, pH 7.5) plus freshly added 0.2 mg ml Proteinase K (from a stock of 20 mg/ml proteinase K, 50% v:v glycerol, 10 mM Tris HCl, pH 7.5) and were incubated at 37°C for 30 min. Samples are precipitated at 4°C for 30 min by the addition of 4 ml 10% TCA (10% w:v TCA, 2% w:v $Na_4P_2O7.10H_2O$). 40 μl (1% of 4ml) of the total reaction was spotted on a paper disc. Insoluble material was recovered from solution by filtration through a glass fibre filter mounted on a vacuum manifold. The glass fibre filters were twice washed in 5% TCA (5% w:v TCA, 0.5% w:v $Na_4P_2O7.10H_2O$), once in 100% ethanol and then air dried. The paper and glass fibre filters were then quantified by scintillation counting. Precipitated material was expressed as a percentage of total counts (%TC) from which DNA replication (ng/μl) was calculated by multiplying by a factor of 0.654. The extent of nuclear formation was followed under the light microscope (phase contrast). All incubations were carried out at 23°C.

Licensing factor extract (LFE) was prepared as described (Chong et al., 1997). The initial steps for preparing metaphase extracts were followed. Before the final spin the extract was activated with 0.3 mM $CaCl_2$ for 15 minutes then diluted 5 fold with "Licensing Factor Buffer" (LFB: 40 mM Hepes KOH pH 8.0, 20 mM $K_2HPO_4$/ $KH_2PO_4$ pH 8.0, 2 mM $MgCl_2$, 1 mM EGTA, 2 mM DTT, 10% (w/v) sucrose and 1 μg/ml each of leupeptin, pepstatin and aprotinin) supplemented with 50 mM KCl (i.e. LFB1/50) and spun to remove membrane components at 84,000 x g in a pre-cooled SW55 rotor swinging bucket rotor at 4°C for 40 min. The clarified supernatant was frozen, in aliquots, in liquid nitrogen and stored at -80°C till required.

Extracts immunodepleted for either ORC or Cdc6 were prepared as described (Chong et al., 1997). Briefly, rProtein A agarose beads (GEHC), preincubated with 2 volumes of either anti-Orc1 or anti-Cdc6 serum, were twice incubated with interphase whole egg extract at a ratio of 1 volume extract plus 0.7 volume beads. Twice depleted extract, recovered from the beads, was frozen in aliquots in liquid nitrogen and stored at -80°C.

## Desalted & Apyrase Treated Extracts

Desalted interphase extract was prepared as described (Gillespie et al., 2001), except that that reaction was scaled up and a PD-10 desalting column (GEHC) was used, as per manufacturer's instructions. Briefly, the column was first equilibrated in LFB1/50. 600 μl of metaphase arrested extract, supplemented with only cycloheximide was released into interphase with 0.3 mM $CaCl_2$ for 15 min at 23°C. Activated extract was applied to the column under gravity and after this had completely entered into the resin bed, 2.3 ml of buffer was then applied; all washes and eluates up to and including this stage were discarded. Following this a further 2.4 ml of buffer was applied to the column and the eluate, containing the peak of protein elution, was collected, pooled, frozen in Eppendorf tubes in liquid nitrogen in appropriate aliquots and stored at -80°C. The final extract recovered was diluted 4-fold. All column steps were undertaken at 4°C.

To prepare Apyrase treated extract, metaphase extract supplemented with only cycloheximide was first released into interphase with 0.3 mM $CaCl_2$ for 5 min at 23°C. Following this, the activated extract was treated with 0.01 U/μl of Apyrase (Sigma, Cat. No. A6410) for 10 mins at 23°C prior to sperm addition.

### Chromatin Isolation

Chromatin isolation and immunoblotting from U20S cells were performed using standard techniques (Moreno et al., 2016); extraction of the chromatin-bound fraction was performed by treatment with CSK extraction buffer (10 mM Hepes, pH 7.4, 300 mM sucrose, 100 mM NaCl, 3 mM MgCl2, and 0.5% Triton-X-100) for 10 min on ice. The pellet, containing chromatin-associated proteins, was processed for immunoblotting. All chromatin isolations from *Xenopus* egg extracts performed using either whole, immunodepleted, desalted or apyrase treated extracts were undertaken in low adhesive Eppendorf tubes, as described (Gillespie et al., 2012, 2016). Briefly, reactions were stopped by the addition of 400 μl of ice-cold NIBTX (50 mM KCl, 50 mM Hepes KOH pH 7.6, 5 mM $MgCl_2$, 2 mM DDT, 0.5 mM spermidine 3HCl, 0.15 mM spermine 4HCl, 0.1% Triton X-100). This was underlayered with 100 μl 15% sucrose in NIBTX. The tubes were spun at 6000 x g for 5 min at 4°C in a swinging bucket rotor. The buffer above the sucrose cushion was removed and the surface of the cushion washed with 200 μl NIBTX before removing the cushion down to ~ 15 μl. The tubes were then spun at 13000 x g for 2 min to focus the chromatin pellet and following this, all the buffer was removed. The chromatin pellet was then resuspended in loading buffer and was subjected to immunoblotting by standard techniques using 4–12% Bis-Tris gradient SDS–PAGE gels (Invitrogen) and enhanced chemiluminescence (ECL) detection (SuperSignal West Pico Chemiluminescent). Images were captured using either standard X-ray film or an ImageQuant LAS4000 (Fujifilm) CCD camera imager and CCD camera images were quantified using ImageStudioLite (Licor) software. The total protein content of chromatin was determined by subjecting samples isolated in NIBTX prepared with an additional 20 mM KCl over a 30% sucrose cushion and spun at 6000 x g for 5 min at 4°C in a swinging bucket rotor, to SDS-PAGE and visualising resolved polypeptides by SYPRO-Ruby staining.

The plasmid DNA binding assay was performed as described (Harvey and Newport, 2003) using pET28 as the template. Plasmid samples were diluted in 2 ml of ELB (50 mM KCl, 2.5 mM $MgCl_2$, 10 mM HEPES pH 7.7, 250 mM sucrose) with 0.2 mM ATP and 0.5% Triton X-100, and centrifuged in a pre-cooled SW55 swinging bucket rotor at 100,000 × g at 4°C for 10 min. Chromatin pellets were washed once in ELB-ATP-Triton X-100 and respun. Isolated samples were subjected to SDS-PAGE and immunoblotting and a proportion was run on an EtBr stained 1% agarose gel in 0.5x TBE to assess DNA recovery. Partially purified fractions of ORC were prepared by precipitation of LFE with 4.25% PEG and were resuspended in LFB1/50 supplemented with 2.5 mM ATP (Gillespie et al., 2001).

### Antibodies and Recombinant Protein

Antibodies used for immunoblotting were: anti-Orc1 (Rowles et al., 1996), -Orc2 & -Cdc6 (Oehlmann et al., 2004), -Cdt1 (Tada et al., 2001), -Mcm3 and 7 (Prokhorova and Blow, 2000), -hMCM2 (BM28) (610701; BD Biosciences), -hMCM5 (Sc136366; Santa Cruz), -Lamin B1 (16048; Abcam) and -α-Tubulin (T6199; Sigma-Aldrich). Recombinant geminin-DEL was a gift from Andrew Ferenbach (Ferenbach et al., 2005).

### Polyethylene Glycol Precipitation

ATP-depleted extracts were prepared from LFE by precipitating with 16% (w/v) PEG6000 (using a stock 50% (w/v) PEG solution), incubated on ice for 30 min, and centrifuged a 12,000 x g for 10 min in a fixed-angle rotor. The pellet was resuspended in LFB1/50 at 0.4X and refrozen till required.

### Licensing Assay

Licensing assays were performed essentially as described (Chong et al., 1997). Briefly, sperm were first incubated in drug treated interphase extract for 15 min at 23°C, during which time the DNA has the opportunity to be licensed. 2 volumes of interphase extract containing an excess of geminin-DEL (McGarry and Kirschner, 1998; Tada et al., 2001) and [$\alpha$-$^{32}$P]dATP was then added to the samples. The geminin prevents any further licensing. The extracts were then incubated at 23°C for 90 min to allow DNA replication to be driven by origins licensed in the previous step.

## QUANTIFICATION AND STATISTICAL ANALYSIS

Statistical data for individual experiments are presented in the appropriate figure legends. In all cases “n” is the number of independent experimental repeats from which the mean ±S.E.M. has been calculated.

Cell Chemical Biology, Volume *24*

## Supplemental Information

# The High-Affinity Interaction between ORC and DNA that Is Required for Replication Licensing Is Inhibited by 2-Arylquinolin-4-Amines

**Nicola J. Gardner, Peter J. Gillespie, Jamie T. Carrington, Emma J. Shanks, Stuart P. McElroy, Emma J. Haagensen, Julie A. Frearson, Andrew Woodland, and J. Julian Blow**

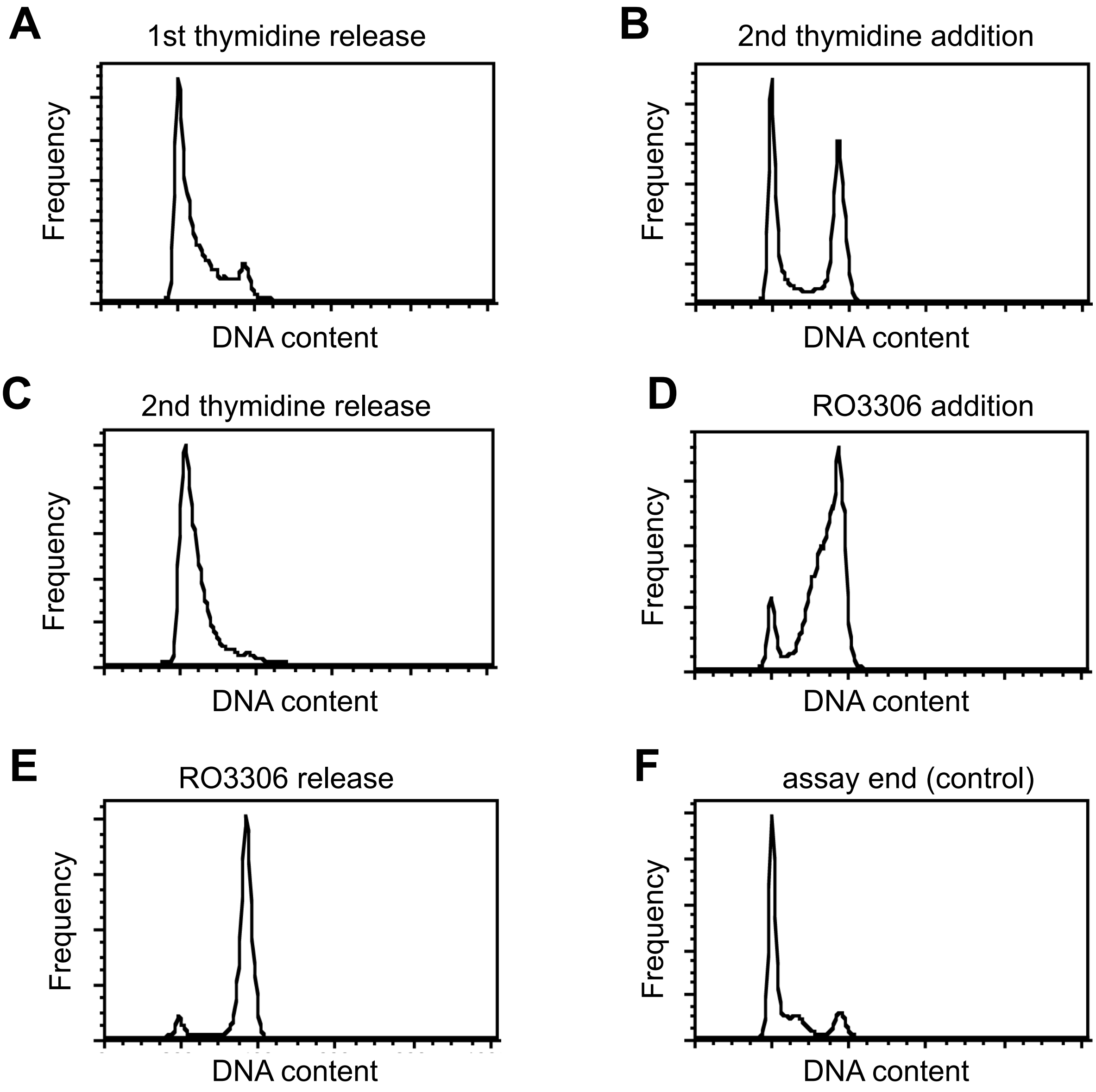


**Supplementary Figure S1.** Flow cytometry analysis of DNA content of cells at different stages in the screen (relates to Figure 1).

Cells were taken through the steps of the screen as shown in Figure 1A. At different steps, cells were isolated and stained with DAPI and their DNA content analysed by flow cytometry. **A.** Cells after the first 16 hr thymidine block. **B.** Cells 12 hr after release from the first thymidine block at the point when the 2nd thymidine block was applied. **C.** Cells at the end of the 2nd(12 hr) thymidine block. **D.** Cells 6 hr after release from the 2nd thymidine block, at the point when RO3306 was added. **E.** Cells after 10 hr RO3306 treatment, at the point of test compound addition. **F.** Cells 8 hr after release from the RO3306 block, at the point when the final Mcm4 assay was typically performed.

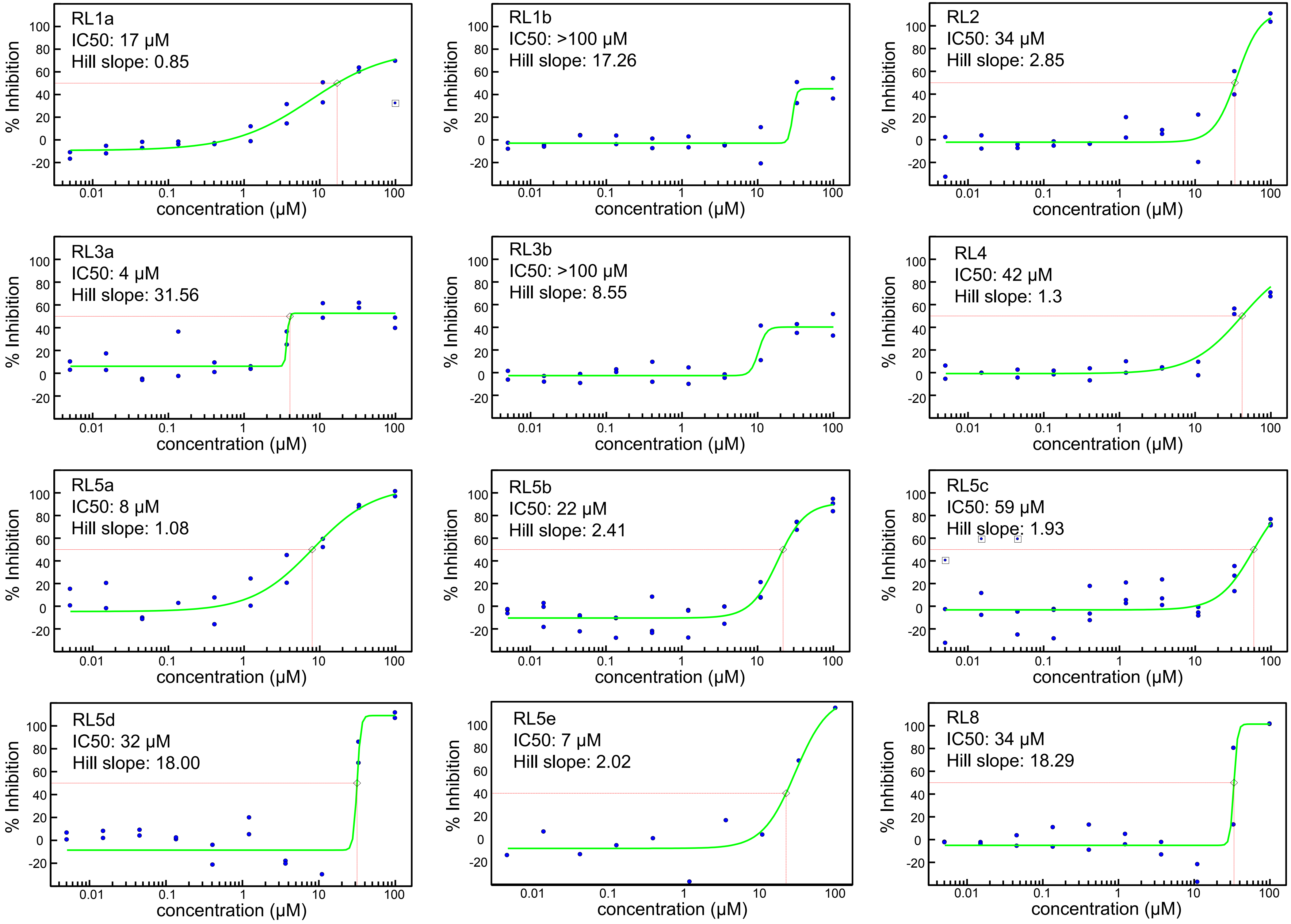


**Supplementary Figure S2.** Titration curves for selected compounds (relates to Figure 2).
12 compounds (RL1a-b, RL2, RL3a-b, RL4, RL5a-e and RL8) were selected after re-screening for a titration analysis. Assays for inhibition of Mcm4 DNA binding were performed in duplicate (apart from RL5e which was performed only once) and used to determine IC50 and Hill slope.

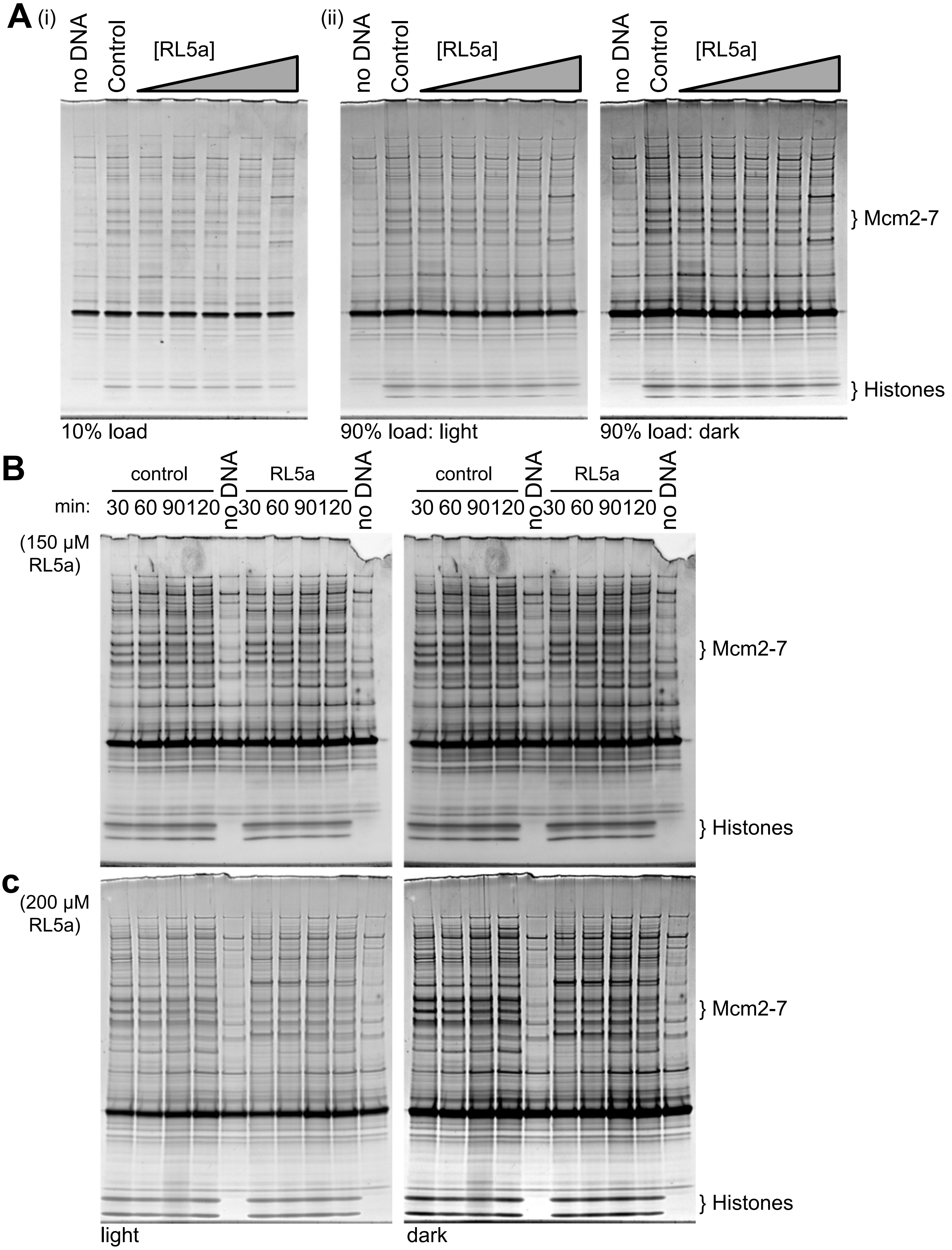


**Supplementary Figure S3.** SYPRO Ruby stained SDS-PAGE gels of chromatin isolated from *Xenopus* egg extract ± RL5a (relates to Figure 3).

Chromatin was isolated from *Xenopus* egg extract, supplemented or not with RL5a, at the indicated times, over a sucrose cushion. Isolated chromatin was subjected to SDS-PAGE and gels were stained with SYPRO Ruby to visualize recovered proteins. A 'no DNA' control was included to facilitate identification of chromatin associated proteins. **A.** Egg extract was supplemented with 25, 50, 100, 150 and 200 μM RL5a and chromatin was isolated at 30 min; (i) 10% load, (ii) 90% load, light and dark exposures. **B, C.** Egg extract was supplemented with RL5a at either (B) 150 μM or (C) 200 μM, and chromatin was isolated at the indicated times. Light and dark exposures of 90% load are shown.

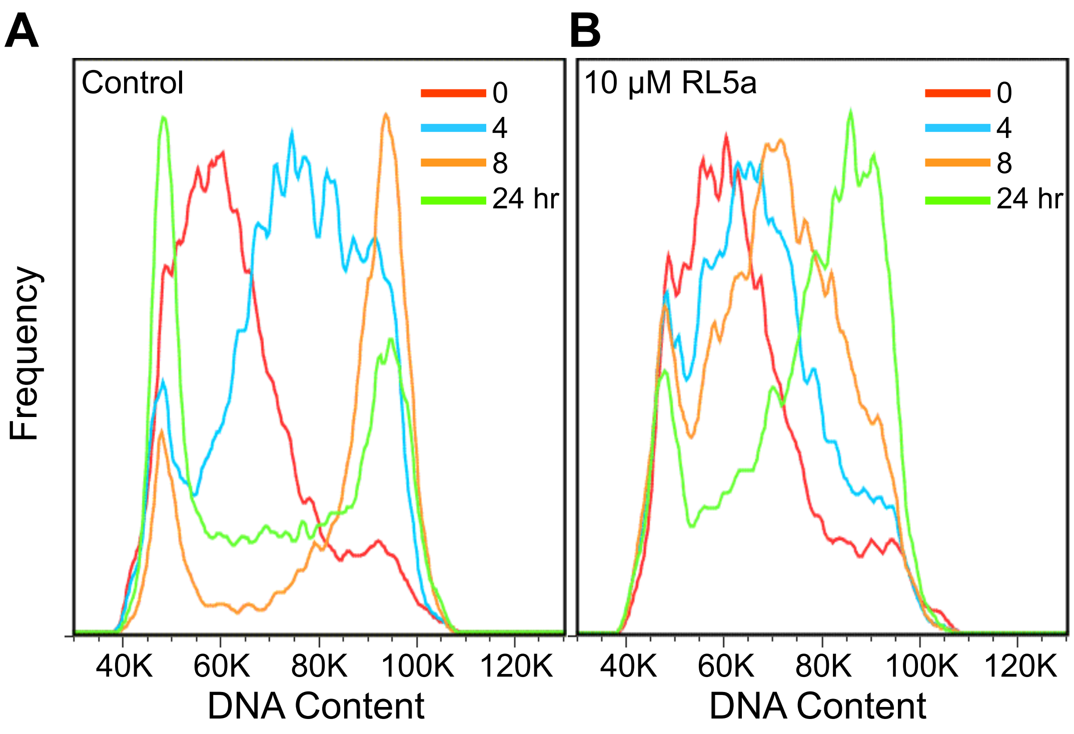


**Supplementary Figure S4.** Effect of RL5a after double-thymidine release (relates to Figure 3).

Following release from a double-thymidine synchronisation, in the absence (A) or presence (B) of 10 µM RL5a, DNA content (PI) of U2OS cells was measured by flow cytometry at the indicated times: 0 hr (red), 4 hr (blue), 8 hr (orange) and 24 hr (green).

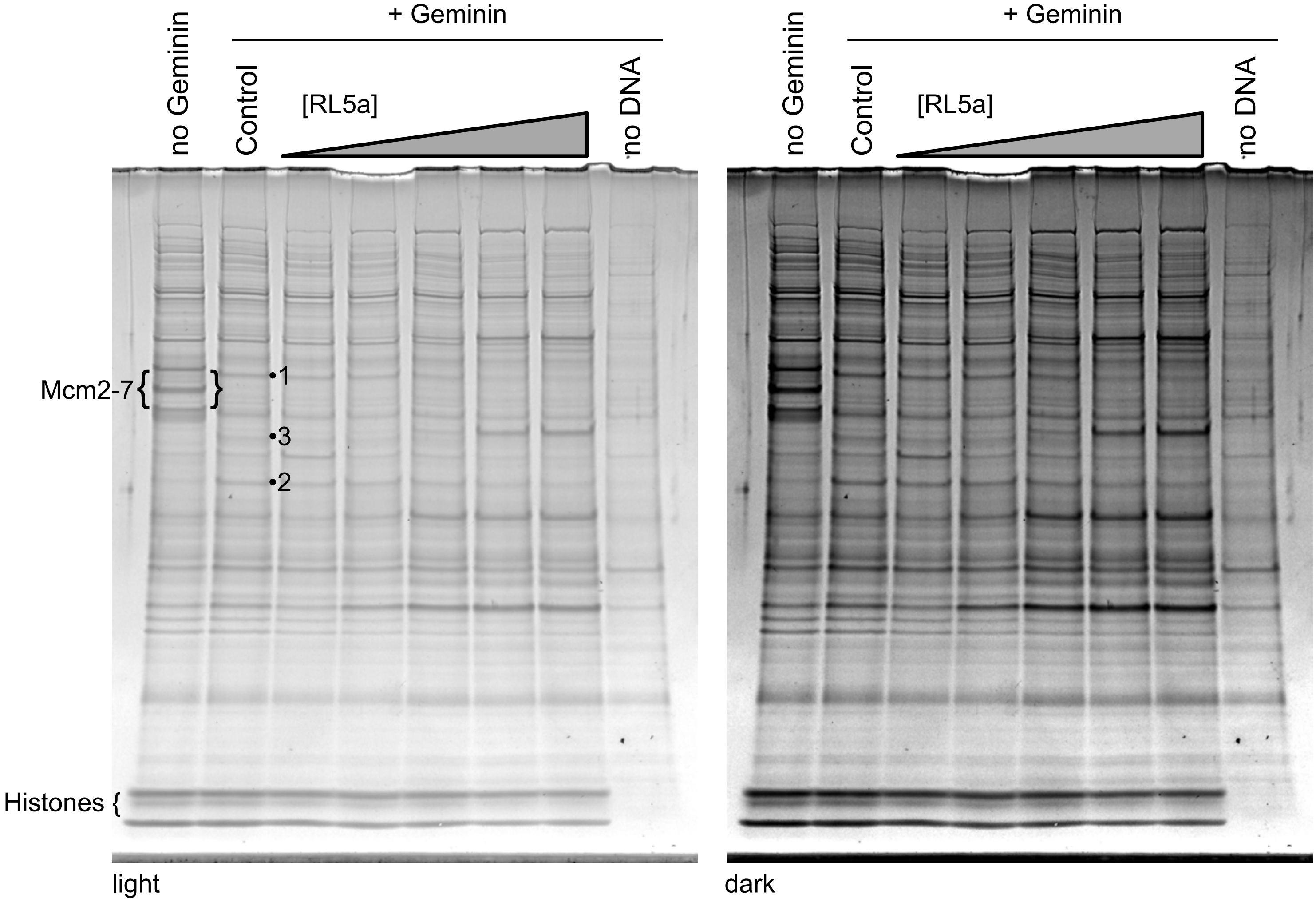


**Supplementary Figure S5.** SYPRO Ruby stained SDS-PAGE gel of chromatin isolated from geminin treated *Xenopus* egg extract ± RL5a (relates to Figure 5).
Chromatin was isolated from *Xenopus* egg extract, treated (or not) with geminin and supplemented (or not) with 25, 50, 100, 150 and 200 μM RL5a, over a sucrose cushion, at 20 min. Isolated chromatin was subjected to SDS-PAGE and the gel was stained with SYPRO Ruby to visualize recovered proteins. A 'no DNA' control was included to facilitate identification of chromatin associated proteins. Light and dark exposures are shown on the left and right respectively. The positions of Mcm2-7({}), Orc1 (•1), Orc2 (•2) and Orc3 (•3) are indicated.

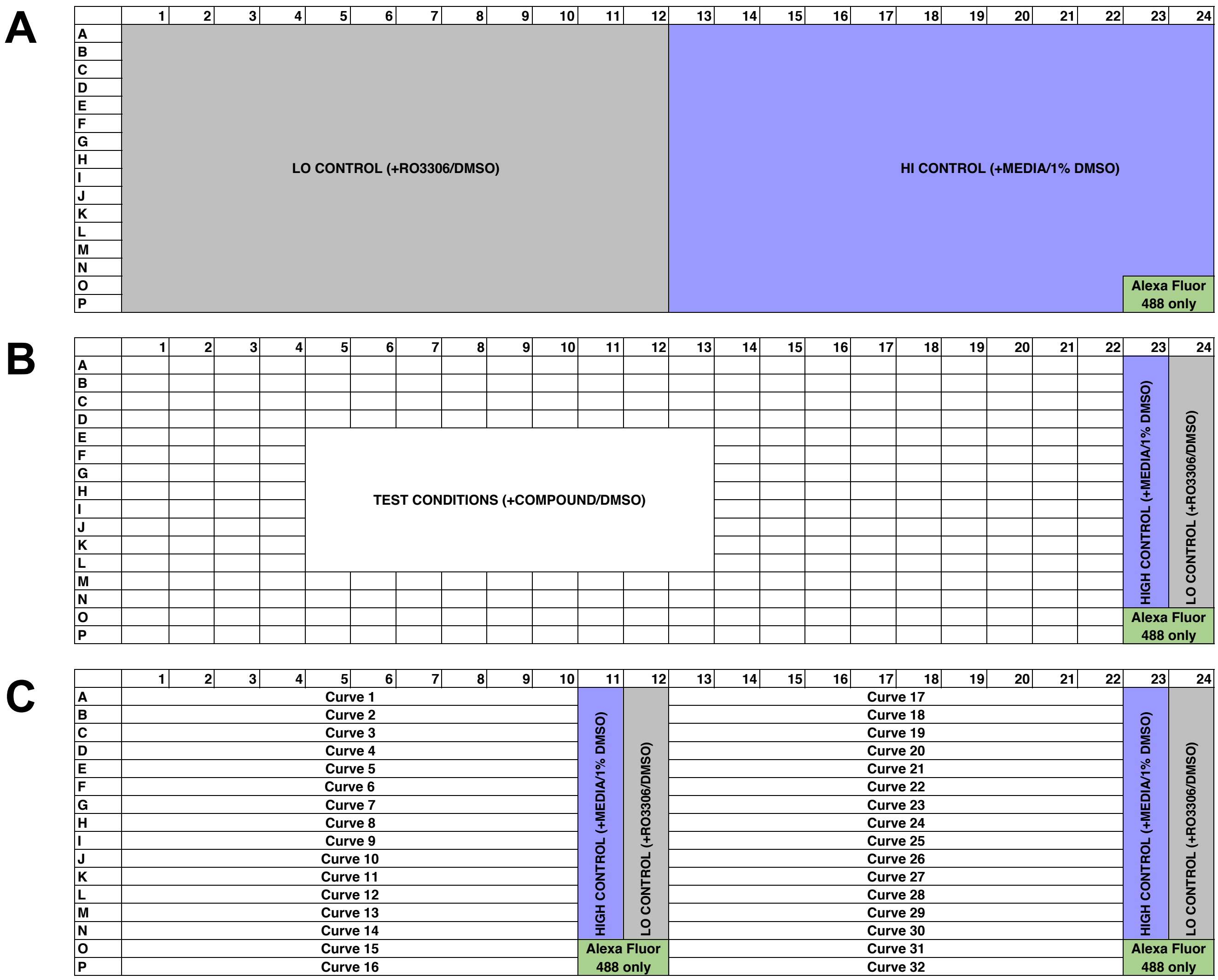


**Supplementary Figure S6.** Screening plate plans (relates to Figures 1 and 2). The plate plans for screening Quality Control plates (A), single point / duplicate point assay plates (B) and potency plates (C) are shown. Grey: Lo control (no release from RO-3306). Purple: Hi Control (release into G1 with DMSO alone). Green: Alexa Fluor 488 only (as Hi control, but no primary antibody). White: test conditions.
